# Mito-xenophagic killing of bacteria is coordinated by a metabolic switch in dendritic cells

**DOI:** 10.1038/s41598-017-04142-5

**Published:** 2017-06-20

**Authors:** Nadine Radomski, Danny Kägebein, Elisabeth Liebler-Tenorio, Axel Karger, Elke Rufer, Birke Andrea Tews, Stefanie Nagel, Rebekka Einenkel, Anne Müller, Annica Rebbig, Michael R. Knittler

**Affiliations:** 1 Institute of Immunology, Friedrich-Loeffler-Institut, Federal Research Institute of Animal Health, Südufer 10, D-17493 Greifswald, Isle of Riems Germany; 2 German Cancer Research Center (DKFZ), Im Neuenheimer Feld 280, D-69120 Heidelberg, Germany; 3 Institute of Molecular Pathogenesis, Friedrich-Loeffler-Institut, Federal Research Institute of Animal Health, Naumburger Strasse 96a, D-07743 Jena, Germany; 4 Institute of Molecular Virology and Cell Biology, Friedrich-Loeffler-Institut, Federal Research Institute of Animal Health, Südufer 10, D-17493 Greifswald, Isle of Riems Germany; 5 University of Tübingen, Interfaculty Institute of Biochemistry, Hoppe-Seyler-Strasse 4, D-72076 Tübingen, Germany

## Abstract

Chlamydiae are bacterial pathogens that grow in vacuolar inclusions. Dendritic cells (DCs) disintegrate these compartments, thereby eliminating the microbes, through auto/xenophagy, which also promotes chlamydial antigen presentation via MHC I. Here, we show that TNF-α controls this pathway by driving cytosolic phospholipase (cPLA)2-mediated arachidonic acid (AA) production. AA then impairs mitochondrial function, which disturbs the development and integrity of these energy-dependent parasitic inclusions, while a simultaneous metabolic switch towards aerobic glycolysis promotes DC survival. Tubulin deacetylase/autophagy regulator HDAC6 associates with disintegrated inclusions, thereby further disrupting their subcellular localisation and stability. Bacterial remnants are decorated with defective mitochondria, mito-aggresomal structures, and components of the ubiquitin/autophagy machinery before they are degraded via mito-xenophagy. The mechanism depends on cytoprotective HSP25/27, the E3 ubiquitin ligase Parkin and HDAC6 and promotes chlamydial antigen generation for presentation on MHC I. We propose that this novel mito-xenophagic pathway linking innate and adaptive immunity is critical for effective DC-mediated anti-bacterial resistance.

## Introduction

Chlamydiae are Gram-negative obligate intracellular bacteria that infect mainly epithelial mucosae, causing a broad spectrum of diseases in humans and animals^1^. Within membrane-bound vacuoles called inclusions, they undergo a biphasic developmental cycle alternating between infectious, but metabolically inactive elementary bodies (EBs) and non-infectious metabolically active reticulate bodies (RBs)^1^. *Chlamydia psittaci* is the causative agent of psittacosis, a widespread infection in psittacine birds and domestic poultry^1^. Zoonotic disease transmission of the microbe to humans has also been reported^2^, leading to life-threatening pneumonia with systemic bacterial spread, myocarditis, hepatitis, and encephalitis^1^. *C. psittaci* is regularly detected in non-avian domestic animals as well as in rodents and wildlife^1^. Non-avian strains can cause abortion and chronic obstructive pulmonary disease^1^.

Chlamydiae induce cell-mediated immune responses in humans and mice^3^. Such immune responses are initiated by dendritic cells (DCs), which perform a sentinel function by internalizing antigens in peripheral tissues. Within secondary lymphoid organs, DCs then process and display these antigens on surface MHC molecules to stimulate CD4^+^ and CD8^+^ T cells. DCs are among the first professional antigen presenting cells (APCs) encountered by chlamydia^4^, and cytotoxic CD8^+^ T cells, primed by infected DCs, likely play an important role in the effective anti-chlamydial immune response^3^. However, the mechanisms by which chlamydial antigens are processed for MHC I presentation are poorly understood.

Autophagy mediates the lysosomal degradation of cytosolic material including protein aggregates (aggrephagy) and damaged mitochondria (mitophagy). To achieve this, a membrane called phagophore engulfs cytosolic content and isolates it into a sealed double membrane-bound autophagosome. This then matures along the endocytic pathway before fusing with lysosomes^5^. Autophagy is also an important defence mechanism that functionally links to downstream activation of the innate and adaptive immune system^5^. Selective autophagosomal degradation of foreign microbes, termed “xenophagy”, is involved in the degradation of bacteria located in the cytosol and in vacuolar compartments. The molecular mechanisms underlying cargo selection and regulation of autophagy and xenophagy are only partly understood, but likely rely on cargo-specific receptors on autophagic membranes^5^.

We previously established a mouse model for non-avian *C. psittaci* infection^6^ and identified an autophagy-dependent immune defence pathway in DCs, in which chlamydial antigens are generated via autophagosomal degradation of cytosolically released microbes following host-mediated disruption of their inclusions^6^. Here, we unravel how infected DCs destabilise chlamydial compartments by metabolic switch and use mito-xenophagy to degrade this material for MHC I cross-presentation. We further identify a TNF-α/cPLA2/AA axis involved in regulating this pathway and the components of the autophagy machinery responsible for executing this process.

## Results

### Dendritic cell-derived TNF-α drives cPLA2-dependent disruption and autophagic clearance of chlamydial compartments

**Figure 1:**
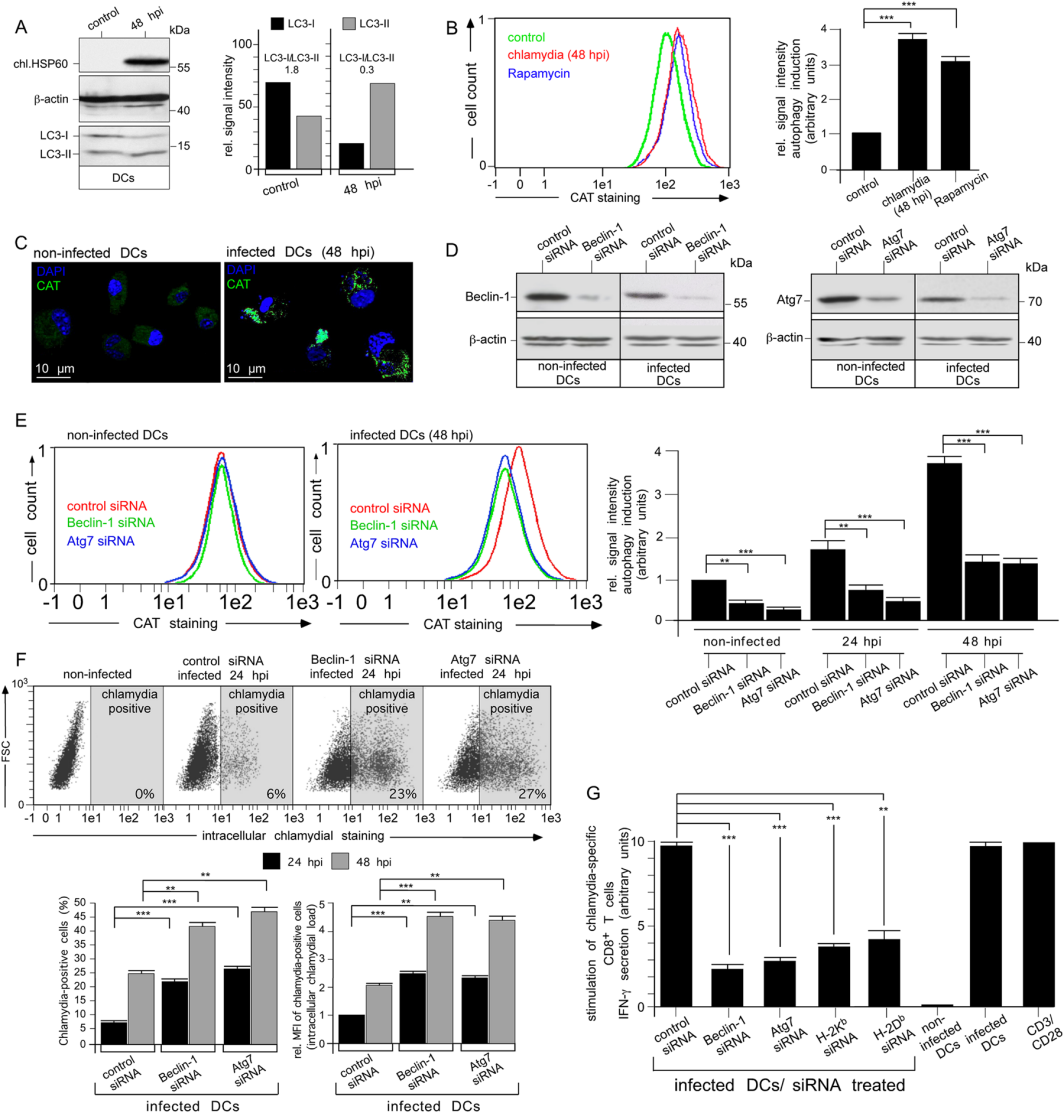
Enhanced autophagy in chlamydia-infected DCs is critical for intracellular degradation and MHC I-presentation of bacteria. (**A**) Chlamydia-infected DCs were analysed in Western blots using antibodies against chlamydial (chl.) HSP60, LC3, and β-actin (left). The LC3-I:LC3-II ratio was determined by densitometric scanning (right panel). (**B**,**C**) Autophagosome formation in DCs was analysed using CytoID labelling (fluorescent cationic amphiphilic tracer CAT) monitored with flow cytometry (left and right, Rapamycin was used as autophagy inducer) (**B**) or fluorescence microscopy (CAT is shown in green, DAPI in blue) (**C**). (**D**) Western blot analysis of infected (48 hpi) and non-infected DCs silenced for Beclin-1 and Atg7. (**E**) Beclin-1- and Atg7-silenced DCs as well as control cells were infected or not and analysed by CytoID staining. The left and middle panel show representative flow cytometry experiments of non-infected and infected (48 hpi) DCs. Data from three independent experiments (non-infected, 24 and 48 hpi) are summarised as bar graphs with arbitrary units (right). Data in (**B** and **E**) were normalised such that the value obtained for non-infected cells (control siRNA in (**E**) was set to 1. (**F**) Beclin-1- and Atg7-silenced DCs were infected or not with chlamydia and analysed by flow cytometry using the IMAGEN kit. The upper panel shows a representative analysis at 24 hpi. Data from three independent experiments (24 and 48 hpi) are summarised in bar graphs (lower panel). The number of chlamydia-positive cells was measured, as well as the bacterial load (MFI) of infected cells. MFIs obtained for infected cells (24 hpi) were set to 1 arbitrary unit. (**G**) DCs were siRNA-silenced for Beclin-1 and Atg7, MHC I (H-2K^b^ and H-2D^b^) and then infected with chlamydia. AllStars siRNA, non-infected cells and anti-CD3/CD28-beads were used in control experiments. DCs were cocultured with chlamydia-sensitised CD8^+^ T cells. IFN-γ secreted by CD8^+^ T cells was assayed by ELISA. Relative values of IFN-γ secretion are expressed in arbitrary units (maximum value was set to 10) and are means ± SD of three independent experiments. Statistical analysis in (**B**,**E**,**F**,**G**) was performed as described in Methods (**p < 0.01; ***p < 0.001 versus controls; n = 3).

By using C57BL/6 mice, JAWSII cells (an established BM-derived mouse DC line with homogeneous and consistent cell culture properties)^7^ and the non-avian *C. psittaci* strain DC15^8^ as a model system for infection, we could demonstrate that chlamydia from structurally disintegrated inclusions are targeted for autophagy and the generation of MHC I-presented peptide antigens^6^. Based on this, we proposed that autophagy constitutes a critical pathway in the intracellular defence against chlamydia in infected DCs. Indeed, chlamydial infection induces autophagy in DCs, as shown by LC3-I-to-LC3-II conversion (Fig. 1A) and autophagy-specific Cyto-ID Green labelling (Fig. 1B,C). This induction was substantially reduced by knockdown of critical autophagy factors such as Beclin-1 and Atg7 (Fig. 1D,E). Strikingly, interference with autophagy drastically increased both the number of chlamydia-positive DCs as well as their bacterial load (Fig. 1F). Moreover, autophagy-impaired DCs displayed poor stimulation of chlamydia-specific CD8^+^ T cells (Fig. 1G). It should be noted that during the course of the respective antigen presentation experiments (48 hpi), siRNA-mediated silencing of Beclin-1 and Atg7 did not affect expression and/or infection-dependent induction of surface MHC I (H-2K^b^ and H-2D^b^), CD80, CD86, PD-L1 or PD-L2. Thus, in flow cytometry studies (Suppl. Fig. S1A,B and C) no measureable differences were observed for surface MHC I and coregulatory molecules of infected and non-infected DCs before and after knockdown of the two autophagy factors. The same was also true for infection-induced TNF-α secretion of the DCs. Results from ELISA experiments (Suppl. Fig. S1D) revealed no detectable differences between infected and non-infected DCs before and after Beclin-1 and Atg7 silencing. This suggests that the reduced CD8^+^ T cell stimulation by autophagy factor-silenced DCs is clearly not caused by defective surface expression of MHC I or coregulatory molecules, or disturbed TNF-α secretion required for functional DC maturation. Taken together, it is tempting to assume that the reduced stimulation of chlamydia-specific CD8^+^ T cells by autophagy-impaired DCs is due to disrupted autophagic generation of chlamydial antigens.

**Figure 2:**
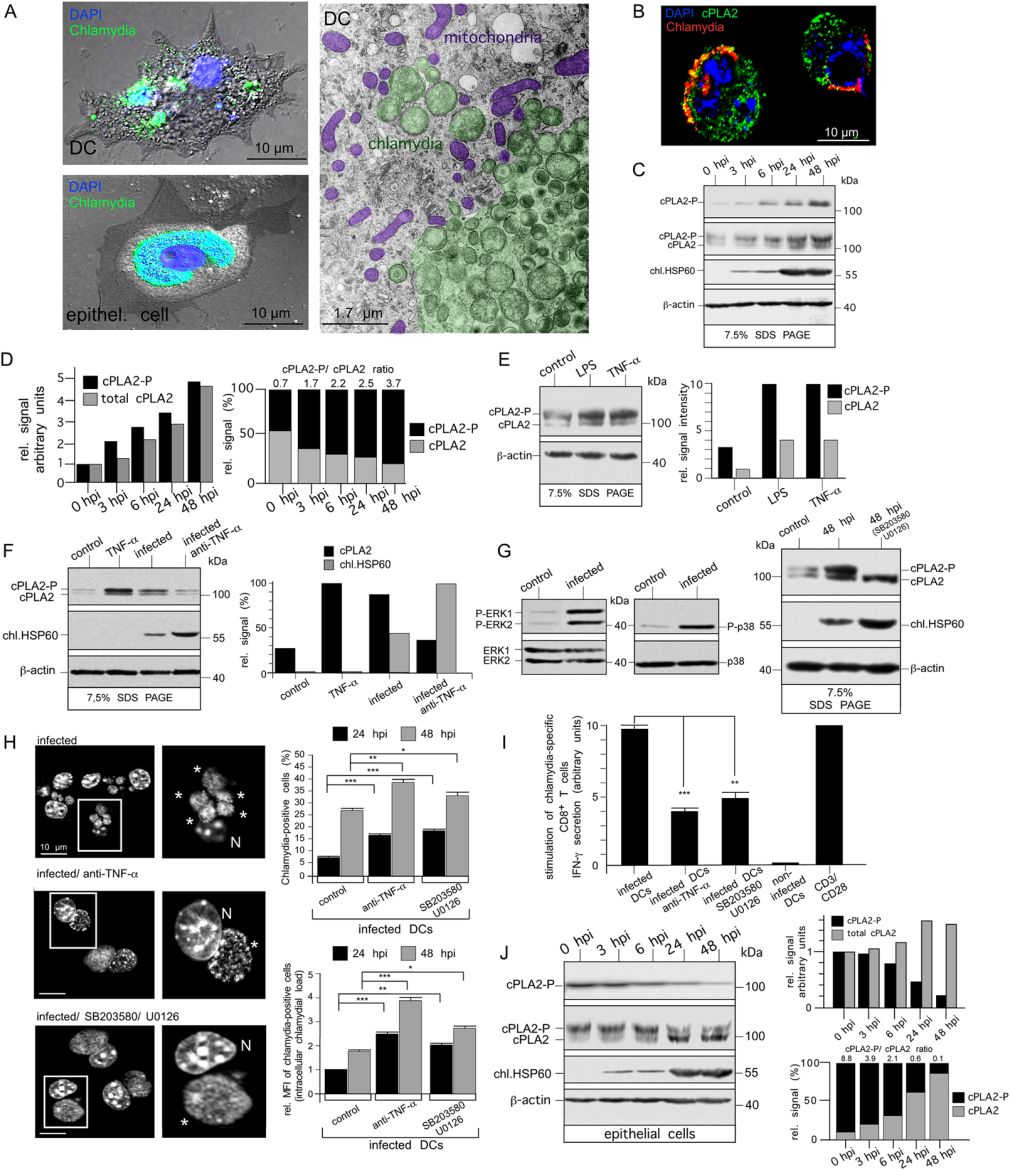
Chlamydial infection of DCs is accompanied by induced expression and activation of AA-producing cPLA2. (**A**) Immunofluorescence of infected DCs (JAWSII) and epithelial cells (MN-R) (48 hpi) (left) stained for chlamydial LPS (green) overlaid on phase-contrast images. Host DNA and intracellular chlamydiae are visualised with DAPI (blue). Electron micrographs of infected DCs (right). Chlamydiae and mitochondria are artificially coloured. (**B**) Immunofluorescence of infected DCs costained for cPLA2 (green) and chlamydial LPS (red) (blue, DAPI). (**C**,**D**) Western blot of DCs infected for 0, 3, 6, 24, or 48 h using antibodies against phospho-cPLA2 (cPLA2-P), total cPLA2, chl.HSP60, and β-actin (**C**). cPLA2-P migrates more slowly on 7.5% SDS-gels than cPLA2. cPLA2 levels and cPLA2-P:cPLA2 ratios were determined by densitometric scanning (**D**). (**E**) Western blot of DCs treated with LPS (10 µg/ml) or TNF-α (30 ng/ml) for 48 h using antibodies against cPLA2 and β-actin. cPLA2-P and cPLA2 levels were determined by densitometric scanning (**D**). (**F**) Western blot of infected DCs treated with TNF-α-neutralizing antibodies using cPLA2, chl.HSP60, and β-actin-specific reagents (left). Quantification by densitometric scanning (right). (**G**) Western blot of infected DCs (12 hpi) using antibodies against phospho-ERK1/2 (P-ERK1/2), total ERK1/2, phospho-p38 (P-p38), and total p38 (left). Western blot of infected DCs treated with ERK1/2 (U0126)- and p38 (SB203580)-specific inhibitors using antibodies against cPLA2, chl.HSP60, and β-actin (right). (**H**) Microscopy of DAPI-stained infected DCs treated with TNF-α-neutralizing antibodies or MAPK inhibitors. Inclusions and disintegrated chlamydial structures are indicated by asterisks (left). Flow cytometry of such treated cells using the IMAGEN kit. Data from three independent experiments (24 and 48 hpi) are summarised in bar graphs as in Fig. 1F (right). (**I**) IFN-γ secretion by chlamydia-specific CD8^+^ T cells stimulated with infected DCs treated with anti-TNF-α or MAPK inhibitors. Results are depicted as in Fig. 1G. (**J**) Western blot of epithelial cells infected for 0, 3, 6, 24, or 48 h using antibodies against cPLA2-P, total cPLA2, chl.HSP60, and β-actin (left). cPLA2 levels and cPLA2-P:cPLA2 ratios were determined by densitometric scanning (right). Statistical analysis in (**H** and **I**) was performed as described in Methods (*p < 0.05; **p < 0.01; ***p < 0.001 versus controls; n = 3).

In epithelial cells, chlamydiae formed large, juxtanuclear inclusions, while in DCs (JAWSII) multiple small (≤5 µm) peripheral compartments were observed (Fig. 2A, left panels). This latter intracellular scenario was also seen in chlamydia-infected primary BMDCs isolated from C57BL/6 mice (Suppl. Fig. S2A) suggesting, in agreement with previous findings^9^, that immortalised and primary BMDCs display a comparable cellular response to intracellular chlamydia. Based on this finding, we continued our studies with the DC line, which provides a more tractable experimental system, and confirmed key-interactions with auto/xenophagy factors in infected primary BMDCs in accompanying immunofluorescence experiments (Suppl. Fig. S2B). Many of the small inclusions in DCs were disrupted with bacteria freely residing in the cytosol (Fig. 2A, right panel). Chlamydial structures and surrounding mitochondria colocalised with the AA-producing enzyme cPLA2^10^ (Figs 2B, 4A and Suppl. Fig. S2B), which was strikingly upregulated and phospho-activated in a time-dependent manner during the course of infection (Fig. 2C,D). LPS and TNF-α enhanced the expression and phosphorylation of cPLA2 (Fig. 2E), while TNF-α neutralization during chlamydial infection impaired the process (Fig. 2F). This suggests a role for DC-derived TNF-α^6^ in cPLA2 activation. The mitogen-activated protein kinases (MAPKs) ERK1/2 and p38 also underwent phospho-activation in response to infection (Fig. 2G, left panel) and their inhibition blocked subsequent phosphorylation of cPLA2. In detail, treatment of infected DCs with p38 inhibitor SB203580 or MEK inhibitor U0126 resulted in inhibition of p38 or ERK1/2, respectively (Suppl. Fig. S3A, B). Each inhibitor caused a partial block of cPLA2 phosphorylation during chlamydial infection (Suppl. Fig. S3A–F), while their combination produced a total block in the phosphorylation of cPLA2 (Fig. 2G, right panel). Restricted growth and structural fragmentation of chlamydial compartments depended largely on TNF-α as well as on p38 and ERK1/2 activity (Fig. 2H, left panels and Supp. Fig. S3E). Consequently, inhibition of these molecules (alone or in combination) resulted in larger numbers of chlamydia-positive cells, higher bacterial load (Fig. 2H and Fig. S3D), and reduced T cell activation (Fig. 2I and Fig. S3F). Our findings support the hypothesis that TNF-α-mediated regulation of cPLA2 is a multifactorial process that is controlled through signalling pathways involving both ERK1/2^11^ and p38^12^. It has been suggested that both MAPKs might act redundantly in phosphorylating cPLA2^13^. TNF-α mediated cPLA2 activation is also preceded by induction of the JNK (c-Jun N-terminal kinase) pathway^14^ (Suppl. Fig. S3G), which, however, probably does not contribute to cPLA2 phosphorylation^11^, but might be involved in the TNF-α-controlled induction of cPLA2 expression^15^. In accordance with these previous reports, we found that infection-induced cPLA2 phosphorylation was indeed unaltered in DCs treated with SP600125 (Suppl. Fig. S3H), which efficiently inhibits JNK activity (Suppl. Fig. S3G). Although in infected DCs cPLA2 expression was slightly decreased in the presence of SP600125 (Suppl. Fig. S3H), the inhibitor did not interfere with inclusion fragmentation (Fig. S3I), increase the bacterial load (Suppl. Fig. S3J) or abolish T cell activation (Suppl. Fig. S3K). Taken together, this suggests that in contrast to ERK1/2 and p38, JNK apparently does not play a critical role in the anti-chlamydial self-defence of infected DCs.

In contrast to DCs, no cPLA2 induction/activation was observed in infected epithelial cells (Fig. 2J). It seems that in epithelial cells the amount of phospho-activated cPLA2 is reduced during infection (24 and 48 hpi).

**Figure 3:**
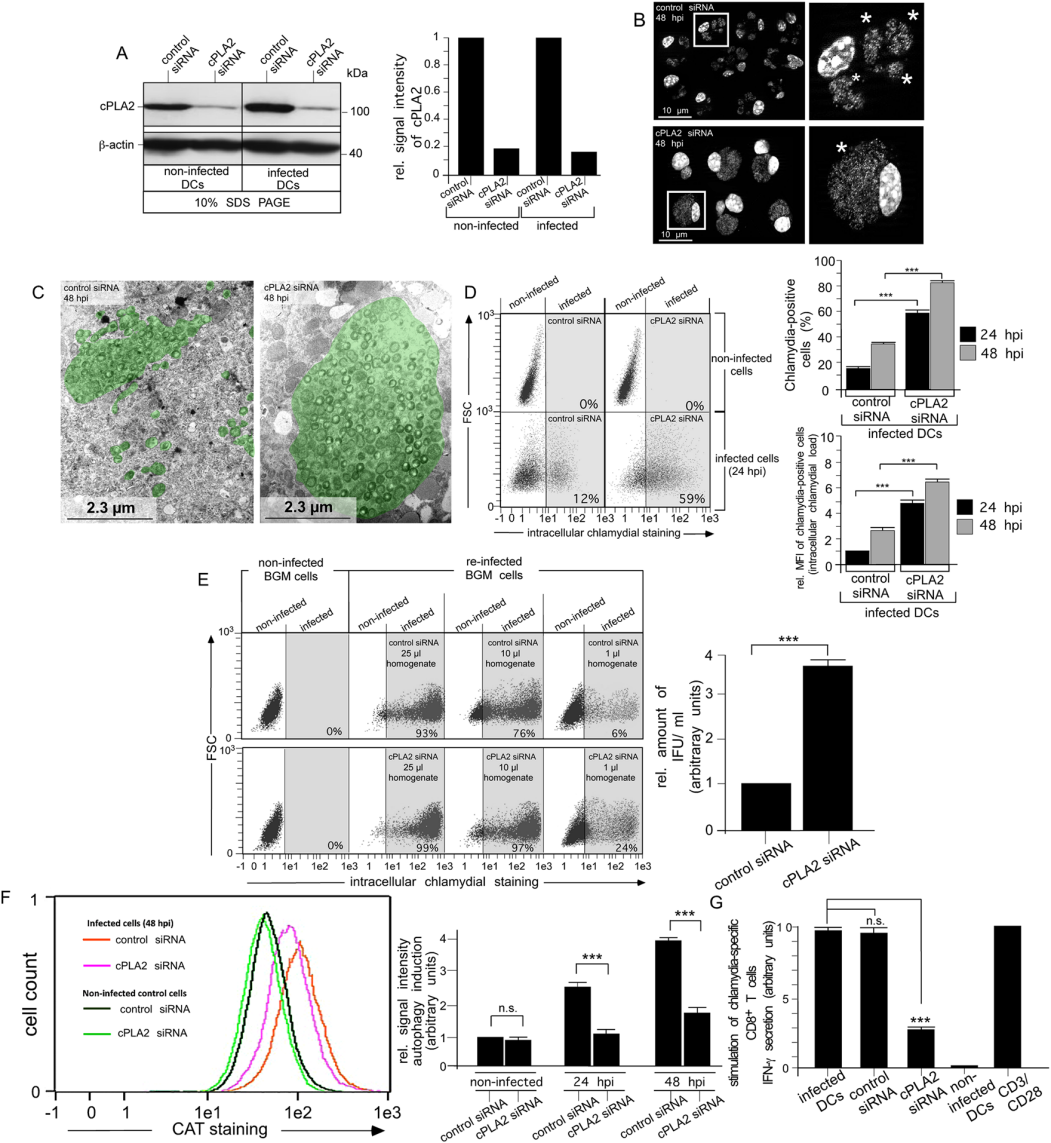
Silencing of cPLA2 interferes with inclusion breakdown and infection-induced autophagy in DCs. (**A**) cPLA2-silenced DCs were incubated or not with chlamydia for 48 h. siRNA silencing was demonstrated by densitometrically analysed Western blots (left and right, cPLA2 is visible as single band on 10% SDS gels) (**B**) cPLA2-silenced and control DCs were infected or not with chlamydia. Nuclei and inclusions were labelled with DAPI. Chlamydial structures are indicated by asterisks. (**C**) Electron photomicrographs of siRNA-treated infected DCs (48 hpi) (control siRNA, left and cPLA2 siRNA, right) Chlamydia and inclusions are artificially coloured green. (**D**) Infected cells were analysed by flow cytometry using the IMAGEN kit. The left panel shows a representative analysis at 24 hpi. Summarised Data (right) from three independent experiments (24 and 48 hpi) show the number of chlamydia-positive cells, as well as the bacterial load (MFI) of infected cells. The MFI for infected cell cultures (24 hpi) was set to 1 arbitrary unit. (**E**) DCs were infected for 48 h. Cell homogenates were titrated on BGM cells (left) to determine the number of recoverable infectious chlamydia. IFU as the measure of infectivity were determined by flow cytometry using the IMAGEN kit. IFU/ml obtained for siRNA-treated control cells was set to 1 arbitrary unit. Pooled data from three separate experiments are shown. (**F**) Cells siRNA-silenced for cPLA2 were incubated or not with chlamydia for 24 and 48 h. Autophagy induction was analysed by using CytoID staining (CAT). The left panel shows a representative flow cytometry analysis (48 hpi). Summarised data from three independent experiments (non-infected, 24 and 48 hpi) are shown as bar graphs with arbitrary units (right). Data were normalised such that the value obtained for non-infected cells (control siRNA) was set to 1. (**G**) DCs were siRNA-silenced for cPLA2 and incubated or not with chlamydia. DCs were cocultured with chlamydia-sensitised CD8^+^ T cells for 48 h. IFN-γ secretion by CD8^+^ T cells was assayed by ELISA. Results are depicted as in Fig. 1G. Statistical analysis in (**D**–**G**) was performed as described in Methods (n.s.: not significant; ***p < 0.001 versus controls; n = 3).

To investigate the functional contribution of cPLA2 in the structural disintegration of chlamydial compartments, we analysed siRNA-silenced DCs (Fig. 3A). Knockdown of cPLA2 caused the establishment of apparently intact, large (≥10 μm) perinuclear inclusions similar to those in epithelial cells (Fig. 3B,C). In contrast, control siRNA-treated DCs showed the typical breakdown of chlamydial compartments with multiple peripheral structures and free cytosolic bacteria. Knockdown of cPLA2 increased the number of chlamydia-positive cells and bacterial load in infected DCs (Fig. 3D). Consequently, cPLA2-silenced DCs harboured and released more infectious EBs than control transfected cells (Fig. 3E). Interestingly, cPLA2 silencing also strongly impaired infection-induced autophagy (Fig. 3F) suggesting that cPLA2-mediated destruction of bacterial inclusions is an essential requirement for their downstream autophagic degradation. In line with this, cPLA2 knockdown also resulted in markedly reduced T cell activation (Fig. 3G).

### Anti-chlamydial defence leads to the loss of functional mitochondria and induces a metabolic switch

**Figure 4:**
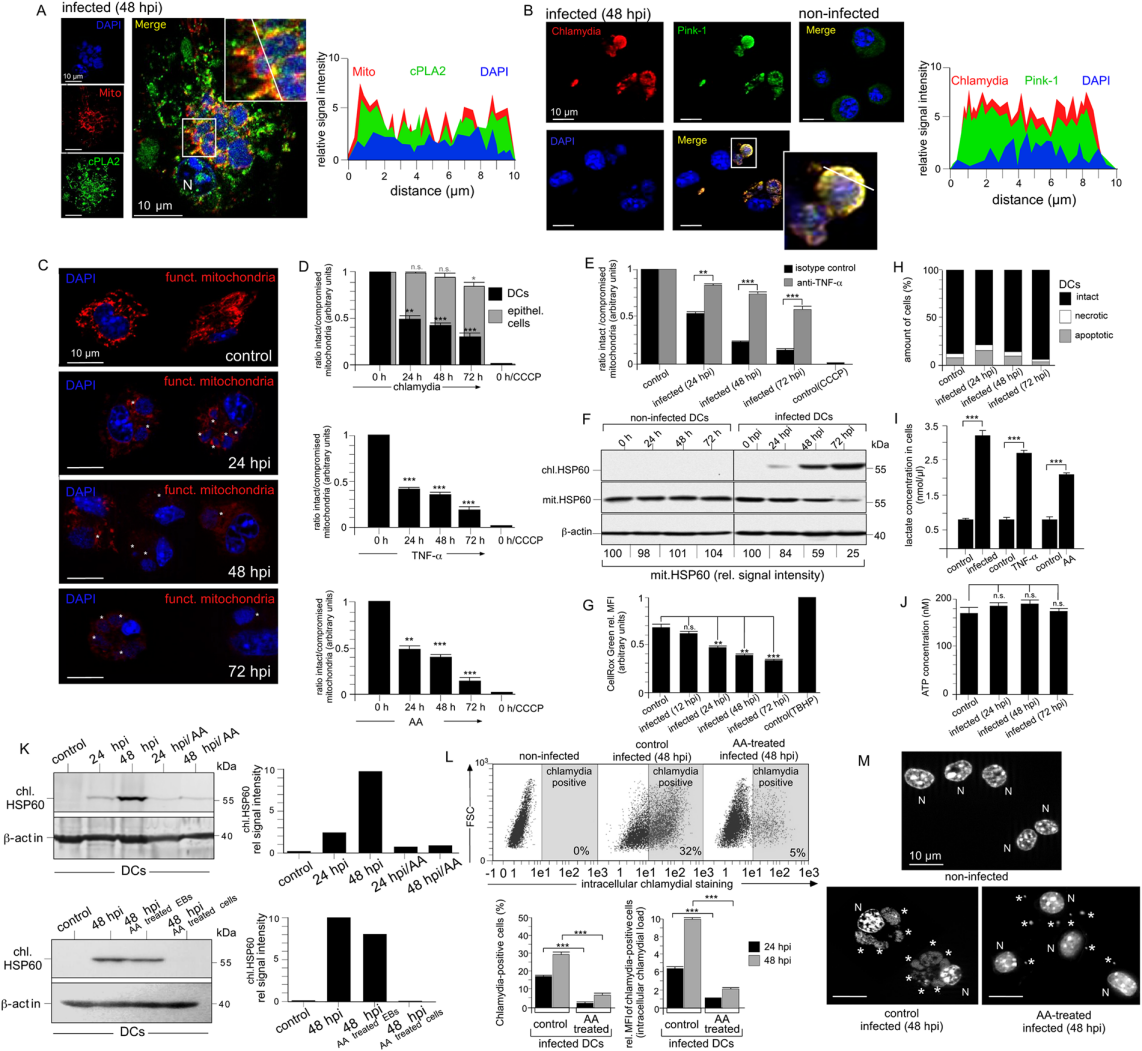
Functional role of AA and metabolic switch in the anti-chlamydial defence of infected DCs. (**A**,**B**) Immunofluorescence of infected DCs stained for cPLA2 (green) and mitotracker (red) (**A**), or Pink-1 (green) and chlamydia (red) (**B**). DNA is visualised with DAPI (blue). Fluorescence intensity along cellular cross sections was measured (ImageJ). Obtained profiles were overlaid and coloured. (**C**) Fluorescence microscopy of infected DCs stained with mitotracker (red) and DAPI (blue). (**D**) DCs (infected (top), treated with TNF-α (middle) or AA (bottom) were loaded with JC-1 and analysed by flow cytometry. The ratio of JC-1 oligomers (intact mitochondria) to JC-1 monomers (compromised mitochondria) is shown as bar diagram. CCCP-treated cells were used as control. (**E**) DCs were pretreated with neutralizing TNF-α antibodies and analysed as in Fig. 4D. (**F**) Western blot of infected and non-infected DCs using antibodies against chl.HSP60, mit.HSP60, and β-actin. (**G**) ROS production of infected DCs was measured by cellROX green using flow cytometry. TBHP-treated cells were used as control. (**H**) Infected DCs stained with Annexin V-FITC and propidium iodide were analysed by flow cytometry. (**I**) Infected, TNF-α- or AA-treated (100 µM) DCs were cultured for 48 h and cellular lactate was determined. (**J**) ATP levels were measured. In Fig. 4D, (**E** and **G**) data are expressed in arbitrary units as mean ± SD for three individual experiments. Maximum values were set to 1. (**K**) Western blot of infected DCs treated with AA 6 hpi before culture for 24 or 48 h. Antibodies against chl.HSP60 and β-actin were used (upper left). A similar experiment with cells and EBs pre-treated with AA (8 h) was also performed (lower left). Quantification by densitometric scanning (right). (**L**) Flow cytometry of infected DCs treated with AA as in Fig. 4K. Data from three independent experiments (24, 48 hpi) are summarised in bar graphs as in Fig. 1F (bottom). (**M**) Microscopy of DAPI-stained infected DCs treated with AA as in Fig. 4K. Chlamydial structures are indicated by asterisks. Statistical analysis in (**D**,**E**,**G**,**I**,**J** and **L**) was performed as described in Methods (n.s.: not significant; *p < 0.05; **p < 0.01; ***p < 0.001 versus controls; n = 3).

Pink-1, a marker of damaged mitochondria^16^, selectively accumulated in infected DCs and strongly colocalised, like cPLA2 (Fig. 4A), with disintegrated bacterial structures (Fig. 4B and Suppl. Fig. S2B). Mitotracker staining was progressively lost during infection indicating a breakdown of mitochondrial membrane potential (MMP, Δψm) (Fig. 4C). Time-dependent collapse of Δψm was further confirmed with JC-1 labelling and occurred selectively in infected DCs but not in epithelial cells, and it could be rescued by treating with TNF-α-neutralizing antibodies (Fig. 4D,E). Progressive mitochondrial dysfunction was mimicked in the absence of infection by treatment with TNF-α or AA (Fig. 4D), suggesting that it is controlled by the TNF-α/cPLA2/AA pathway. Mitochondrial HSP60 (mit.HSP60) gradually disappeared in infected, but not in uninfected cells (Fig. 4F), suggesting that dysfunctional mitochondria were removed over time via mitophagy.

While mitochondria are a major source of ROS, mitophagy is a principal mechanism for reducing reactive oxygen species (ROS) generation, which in turn controls apoptosis^17^. Interestingly, we observed a continuous reduction in cellular ROS levels (12–72 hpi) in infected DCs and fewer apoptotic cells over time (72 hpi) (Fig. 4G,H). This suggests that elimination of dysfunctional mitochondria promotes a pro-survival phenotype. In accordance with this, glycolytic lactate production was strongly enhanced, pointing to an upregulation of aerobic glycolysis, which could also be mimicked with TNF-α and AA treatment in the absence of infection (Fig. 4I). Cellular ATP levels were largely unaffected (Fig. 4J) indicating that aerobic glycolytic ATP production compensated for the loss of mitochondrial energy supply. Altogether, it seems that TNF-α/cPLA2/AA signalling triggers a metabolic switch to glycolysis, promoting cell survival.

DCs infected in the presence of AA displayed dramatically reduced accumulation of chlamydial protein, lower numbers of bacteria-positive cells and overall lower bacterial loads (Fig. 4K,L), with AA acting on the host cell and not (or only weakly) on EBs (Fig. 4K, lower panel). Further, the multiple small chlamydial vacuoles in infected DCs dispersed into even smaller bacterial structures (≤1 µm) (Fig. 4M). Thus, the TNF-α/cPLA2/AA pathway seems to drive the dysfunction and degradation of inclusion-associated mitochondria. This is accompanied by the structural disintegration of mitochondria-dependent chlamydial compartments.

### Aggresomal structures form around bacterial remnants and drive subsequent autophagy and MHC I cross-presentation

**Figure 5:**
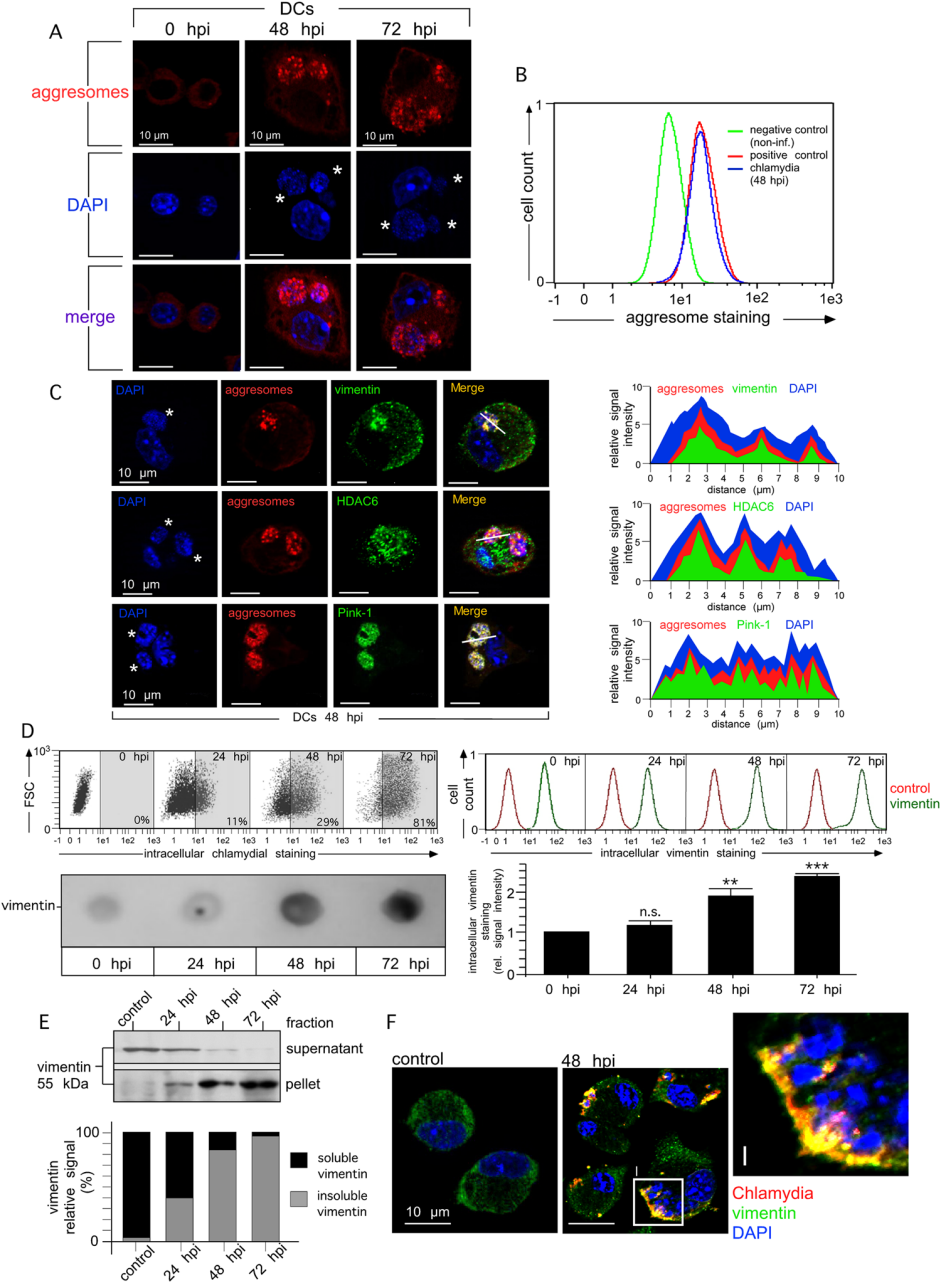
Disintegration of inclusions in DCs is accompanied by vimentin aggregation and aggresome formation around bacterial structures. (**A**) Aggresome formation in DCs (red) was analysed by using the Proteostat aggresome detection kit. DNA was visualised by DAPI (blue). Chlamydial structures are indicated by asterisks. (**B**) For aggresome detection by flow cytometry, DCs were infected or not with chlamydia. Positive control cells were treated with MG-132 for 18 h. Fluorescence profiles of positive control cells, infected and non-infected DCs (48 hpi) were overlaid. (**C**) Infected DCs (48 hpi) were costained for vimentin, HDAC6, Pink-1 (green) and aggresomal structures (red). DNA was visualised by DAPI (blue). The fluorescence intensity along a cellular cross section of interest was measured. Obtained profiles were overlaid and coloured. (**D**) Infected DCs were incubated with IMAGEN kit (upper left panel) or anti-vimentin antibody (upper right panel). Intracellular stainings are depicted as flow cytometry dot plot (upper left panel) and histogram (upper right panel), respectively. Data from the vimentin-staining experiment are summarised by bar graph (lower right panel). The MFI values of non-infected cells (0 hpi) were set to 1 arbitrary unit. A corresponding immuno-dot blot analysis of urea-extracted DCs is depicted in the lower left panel. (**E**) For the analysis of vimentin aggregation, infected DCs were lysed and centrifuged. Pellet and supernatant were analysed for the presence of vimentin in the same Western blot. The signal distribution (%) between soluble and insoluble vimentin was determined by densitometry (bottom panel). (**F**) DCs were infected or not for 48 h and costained for vimentin (green), chlamydial LPS (red) and DNA (blue). Statistical analysis in (**D**) was performed as described in Methods (n.s.: not significant; **p < 0.01; ***p < 0.001 versus controls; n = 3).

Aggresomes accumulated in infected DCs around bacterial structures (Fig. 5A,B and Suppl. Fig. S2B) where they colocalised with Pink-1, the ubiquitin-adaptor protein HDAC6, and the cytoskeletal protein vimentin (Fig. 5C and Suppl. Fig. S2B). Vimentin induction is a characteristic feature of the functional maturation of DCs^18^. In line with this, we observed a massive accumulation of cellular vimentin during infection (Fig. 5D). The protein largely sorted into the insoluble cell fraction, suggesting its association with aggregates (Fig. 5E) and was found in close proximity to disintegrated chlamydial structures (Fig. 5F).

HDAC6 is known to deacetylate α-tubulin in assembled microtubules (MTs)^19^. Unlike in epithelial cells, the protein steadily accumulated during infection in DCs in both the soluble and insoluble cellular fraction, and this was accompanied by progressive loss of acetylated α-tubulin (Suppl. Fig. S4A). While acetylated MTs were highly abundant and clearly associated with chlamydial inclusions in epithelial cells, their localisation was largely restricted to the microtubule organizing centre (MTOC) in infected DCs and they made only few contacts with small chlamydial compartments (Suppl. Fig. S4B,C). Whereas the Golgi apparatus was found in tight association with bacterial vacuoles in epithelial cells, peripheral chlamydial structures in infected DCs appeared spatially remote from this organelle (Suppl. Fig. S4B). Strikingly, in DCs treated with the HDAC6 inhibitor tubacin, chlamydial compartments regained perinuclear localisation and were surrounded by acetylated MTs, very reminiscent of epithelial cells (Suppl. Fig. S4D,E). Moreover, in contrast to the dispersed multiple small chlamydial structures (≤5 µm) in untreated DCs, tubacin caused the appearance of large single inclusions (10–30 µm) (Suppl. Fig. S4E). Thus, HDAC6 activity drives the loss of acetylated MTs, which subsequently are not available for normal structural stabilization, development, and perinuclear transport of chlamydial compartments. This may allow infected DCs to attack destabilised inclusions using additional defence mechanisms.

**Figure 6:**
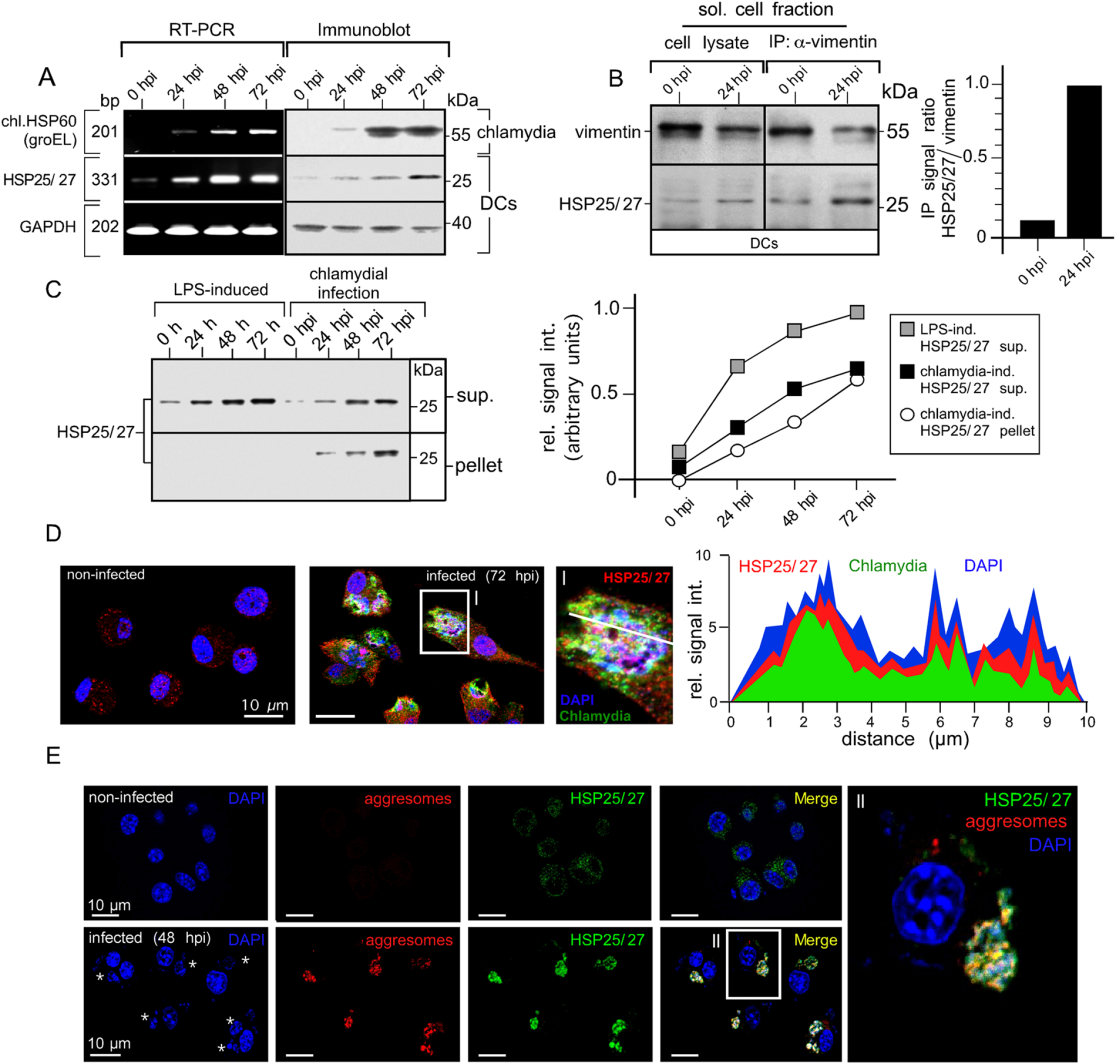
Chlamydial structures interact with HSP25/27 involved in aggresome formation and clearance. (**A**) DCs were infected and total RNA was analysed by semi-quantitative RT-PCR for HSP25/27, chl.HSP60 and GAPDH (left panel). Corresponding cell lysates were analysed in Western blot (right panel). (**B**) DCs were infected or not with chlamydia for 24 h. The soluble fraction of lysates was subjected to immunoprecipitation of vimentin. Western blots were probed for vimentin and HSP25/27 (left panel). Signal quantification was performed by densitometry (right panel). (**C**) DCs were treated with LPS or infected with chlamydia. Cells were disrupted using a Dounce homogeniser and centrifuged. Pellet and supernatant fraction were analysed by Western blot (left panel). HSP25/27 signals were determined by densitometric scanning and plotted in a respective graph as arbitrary units (right panel). (**D**) Non-infected and infected (72 hpi) DCs were costained with antibodies specific for HSP25/27 (red), chlamydial LPS (green) and DNA (blue) (left panel). The enlarged photograph (**I**) shows colocalisation between HSP25/27 and chlamydial structures. The fluorescence intensity along a cellular cross section of interest was measured (right panel). Obtained profiles were overlaid and coloured. (**E**) Infected and non-infected DCs (48 hpi) were costained for HSP25/27 (green), aggresomal structures (red) and DNA (blue). The enlarged photograph (II) shows colocalisation between HSP25/27 and aggresomal structures.

A well-known representative of aggresomal HSPs is HSP25/27 (also termed HSPB1)^20^. HSP25/27 is an ubiquitin-binding chaperone that interacts functionally with different aggresomal components including actin and tubulin, vimentin, Parkin and HDAC6^21^. HSP25/27 was highly up-regulated in chlamydia-infected DCs (Fig. 6A) and displayed enhanced physical interaction with vimentin (Fig. 6B). In agreement with previous studies in different cell types^22–24^, we found that TNF-α does not elevate the protein level of HSP25/27 but controls the phosphorylation of the chaperone (Suppl. Fig. S5A–C). Phosphorylation of HSP25/27 is thought to increase physical complex formation between vimentin and HSP25/27, accompanied by redistribution of vimentin into an insoluble network, by increasing the insoluble/soluble ratio^25^. Indeed, the chaperone also accumulated in the insoluble cellular fraction (Fig. 6C), indicating its association with aggregates. An association with aggregates was not seen in cells merely treated with LPS (a known HSP25/27 inducer)^26^, even though the chaperone was up-regulated in this situation as well (Fig. 6C). HSP25/27 highly colocalised with bacterial compartments (Fig. 6D) and inclusion-associated aggresomes (Fig. 6E). Accordingly, amorphous electron dense structures were found in the vicinity of free cytosolic bacteria, disintegrated inclusions, and intact vacuoles (Suppl. Fig. S6A). Nothing comparable was seen in non-infected DCs. To study whether these structures contained characteristic aggresomal “signature” proteins (HSPs, cytoskeletal and mitochondrial proteins)^27^ as well as chlamydial polypeptides, we analysed isolated aggresomes. Western blots showed a dramatic increase of aggresomal proteins in infected cells and confirmed the presence of known aggresomal components (vimentin, actin, HDAC6, and HSP25/27) as well as chlamydial HSP60 (chl.HSP60) (Suppl. Fig. S6B). Further, liquid chromatography tandem mass spectrometry (nLC MALDI-TOF/TOF MS) demonstrated the presence of actin, vimentin, α/β-tubulin, HSP90, mitochondrial proteins (ATP-synthase α/β, mit.HSP60 and mit.HSP70), as well as various chlamydial polypeptides (chaperones, membrane-bound proteins, enzymes, and ribosomal proteins) (Suppl. Fig. S6C).

**Figure 7:**
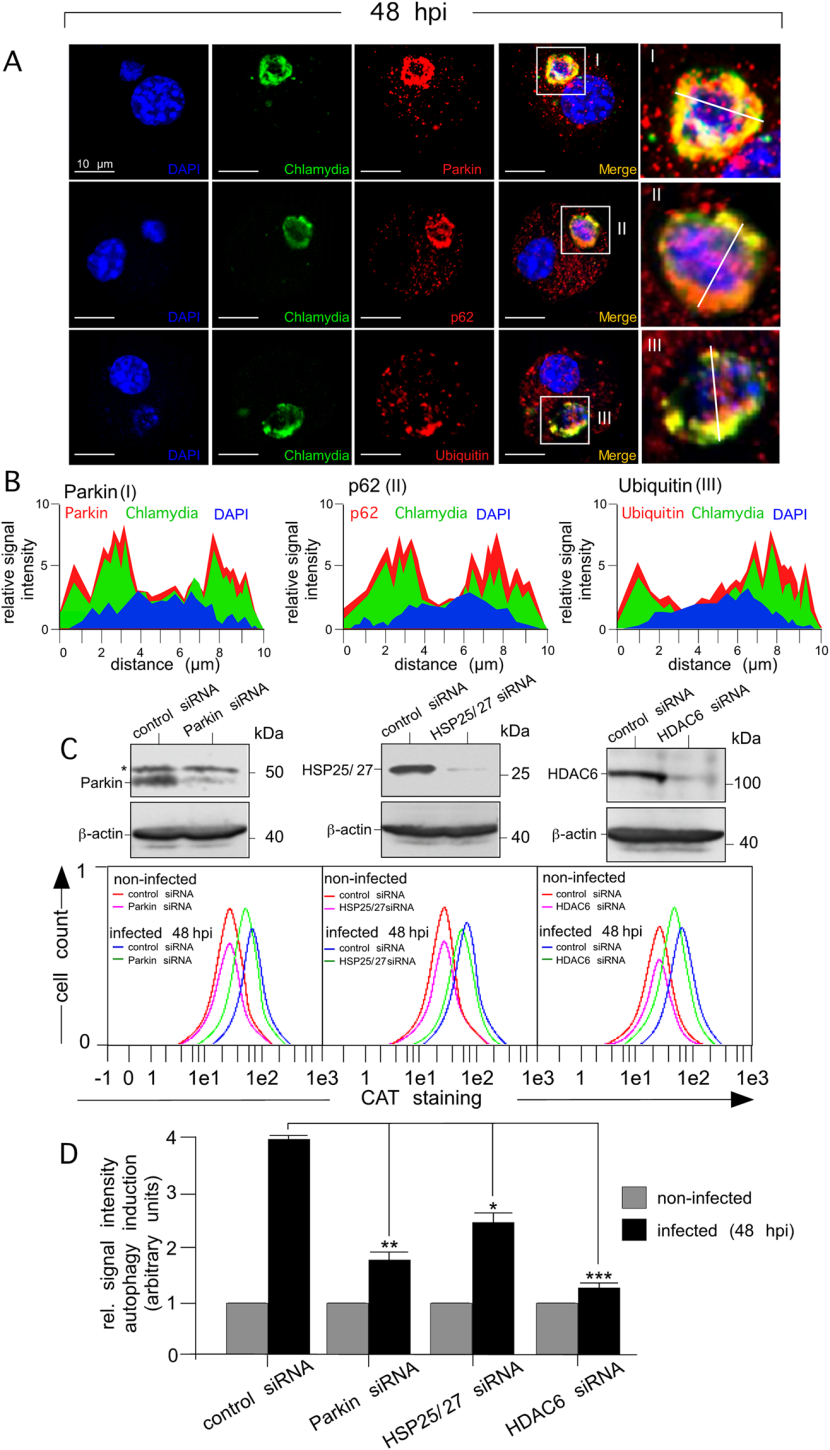
Silencing of aggrephagy-associated proteins affects the autophagosomal degradation of chlamydial structures in infected DCs. (**A**) Infected DCs (48 hpi) were costained for Parkin, p62, or ubiquitin (red), chlamydial structures (green) and DNA (blue) (left panel). Enlarged photographs (I, II and III, right panel) show colocalisation between aggrephagy-associated proteins and chlamydial structures. (**B**) The fluorescence intensity along a cellular cross section of interest was measured. Obtained profiles were overlaid and coloured. (**C**) DCs were siRNA-silenced for Parkin, HSP25/27 and HDAC6 and then infected (48 hpi) with chlamydia. siRNA silencing of Parkin, HSP25/27 and HDAC6 was demonstrated by Western blot (upper panels). Asterisk indicates an unspecific cross-reacting band. Autophagy induction in non-infected and infected (48 hpi) DCs (silenced for the different proteins) was analysed by CytoID staining (CAT). Fluorescence profiles were overlaid to directly compare changes in autophagy (lower panels). Data from three independent experiments (non-infected and 48 hpi) are summarised as bar graphs with arbitrary units (**D**). The value obtained for non-infected cells was set to 1. Statistical analysis in (**D**) was performed as described in Methods (*p < 0.05; **p < 0.01; ***p < 0.001 versus controls; n = 3).

**Figure 8:**
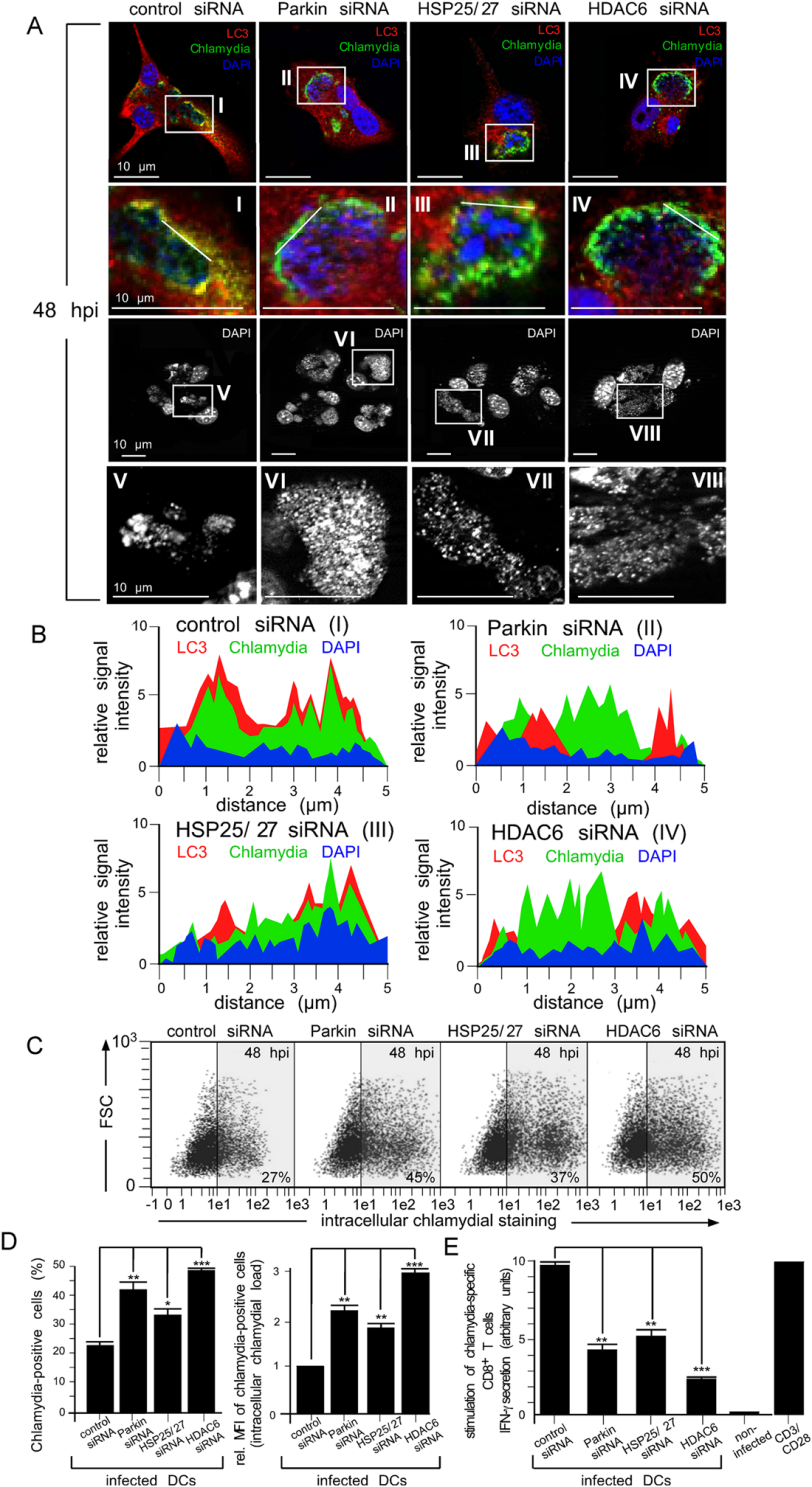
Silencing of aggrephagy-associated proteins increases the bacterial load and affects chlamydial antigen presentation in infected DCs. (**A**) DCs were siRNA-silenced for Parkin, HSP25/27 or HDAC6 and then infected with chlamydia. Cells were fixed and costained for LC3 (red), chlamydial LPS (green) and DNA (blue) (top panel). Enlarged photographs in the second panel from top (I, II, III and IV) show colocalisation between aggrephagy-associated proteins and chlamydial structures. The influence of siRNA silencing on the physical appearance of inclusions was visualised by DAPI (third panel from top). Enlarged photographs (lower panel, V, VI, VII and VIII) display the size and structure of inclusions. (**B**) The fluorescence intensity along a cellular cross section of interest (enlarged photographs I, II, III, and IV in (**A**) was measured. Obtained profiles were overlaid and coloured. (**C**) siRNA-silenced infected DCs (48 hpi) were analysed by flow cytometry using the IMAGEN kit. Summarised data from three independent experiments (**D**) show the number of chlamydia-positive cells, as well as the bacterial load (MFI) of infected cells. The MFI values obtained for infected cell cultures (control siRNA) were set to 1 arbitrary unit. (**E**) DCs were siRNA-silenced for Parkin, HSP25/27 or HDAC6 and infected with chlamydia. AllStars siRNA, non-infected cells and CD3/CD28 beads were used as controls. DCs were cocultured with chlamydia-sensitised CD8^+^ T cells for 48 h. IFN-γ secretion by CD8^+^ T cells was assayed by ELISA. Relative values of IFN-γ secretion are expressed in arbitrary units (maximum value was set to 10) and are the means ± SD of three independent experiments. Statistical analysis in (**D** and **E**) was performed as described in Methods (*p < 0.05; **p < 0.01; ***p < 0.001; n = 3).

HDAC6 as an important stress sensor is thought to facilitate dynein-mediated translocation of Parkin-ubiquitinated substrates and ubiquitin-adaptor proteins to the aggresome^28^. According to this proposed scenario, Parkin, ubiquitin, and ubiquitin-adaptor p62 (also known as sequestosome 1 (SQSTM1)) all colocalised with chlamydial structures in infected DCs (Fig. 7A,B and Suppl. Fig. S2B). Moreover, aggrephagy factors including Parkin, HDAC6, and HSP25/27 were all required for efficient infection-induced autophagy (Fig. 7C,D) and recruitment of LC3 to chlamydial structures (Fig. 8A,B). DCs silenced for any of these three proteins displayed a dramatic enlargement of bacterial vacuoles, occupying almost the entire cell at 48 hpi (Fig. 8A, bottom row). The increase in bacterial load was accompanied by an increase in the number of infected cells (Fig. 8C,D) and a marked reduction in the capability to activate chlamydia-specific CD8^+^ T cells (Fig. 8E). As expected, a triple combination of siRNAs simultaneously targeting Parkin, HSP25/27 and HDAC6 (Suppl. Fig. S7A–E) enhanced the characteristic phenotypes and functional deficiencies that were observed to different extents for each of the respective single knockdowns (Figs 7C,D and 8A–E).

Taken together, our findings strongly suggest that Parkin, HDAC6, and HSP25/27 are critically involved in the autophagosomal clearance and MHC I-presentation of chlamydia in infected DCs.

## Discussion

Various intracellular bacteria depend on host mitochondrial function to parasitize energy and nutrients stores^29^. Chlamydiae, for instance, lack an F1-F0 ATP synthase for H^+^-driven ATP synthesis^30^ as well as genes for the biosynthesis of vitamins, purines, pyrimidines, and almost all amino acids^31^. To import these compounds from the host cell, they express various transporter proteins and ATP/ADP translocases^32^ and are able to recruit mitochondria to their vacuole (e.g. *C. psittaci*) (Figs 2A, 4A)^33^. Although pathogenic chlamydiae may have some limited capacity for independent ATP synthesis^34^, it is unclear to which extent “bacterial-ATP” contributes to their overall energy demands. Studies point to host ATP as the main energy source during the first phase of chlamydial infection, whereas bacterial metabolism might supplement these imports during the growth phase^35^. Antimycin A treatment strongly interferes with the development and metabolism of chlamydia^36^, demonstrating that host cell oxidative phosphorylation is an essential source of ATP for bacterial growth. Moreover, silencing of mitochondrial genes inhibits chlamydial infection^37^. Thus, chlamydiae are strictly dependent on host cell mitochondria and under normoxic conditions the energy provided by glycolytic ATP is not sufficient for the parasite. This is in line with our own results shown in Fig. 4.

We suggest that DCs exploit the bacterial dependency on mitochondria to create an anti-chlamydial intracellular environment destabilizing the pathogen’s vacuoles. Eventually the infected cells disrupt the inclusions through cPLA2 and HDAC6 activity (Figs 2, 3, 4 and 5 and Suppl. Fig. S4) and the energy-depleted and disintegrated chlamydial structures form mixed aggregates with dysfunctional mitochondria (Figs 6, 7 and 8 and Suppl. Fig. S6). These aggregates are finally cleared by autophagic degradation (Figs 1A–E, 3F, 7C,D and Suppl. Fig. S2). We show that this immune defence process is controlled by autocrine TNF-α signalling, which induces and phospho-activates AA-producing cPLA2 via p38 and ERK1/2 (Fig. 2 and Suppl. Fig. S3). Moreover, also the JNK pathway might contribute to some extent to the augmented expression of cPLA2 in infected DCs (Suppl. Fig. S3). TNF-α-driven cPLA2 activation was previously reported in macrophages^38^ where it mediates anti-mycobacterial defence via apoptosis^39^. Further, TNF-α signalling has been associated with mitochondrial dysfunction^40^. TNF-α-induced AA alters the physical properties and dynamics of membranes, affects inner membrane permeability in mitochondria, directly impacts energy coupling, and inhibits cytochrome complexes of the respiratory chain^41^. Thus, AA causes the accumulation of compromised mitochondria with defective oxidative phosphorylation (Fig. 4D)^42^ that are eventually cleared by autophagy^43^. Additionally, cPLA2 and its product drive cell autonomous defence via regulation of immunity-related GTPases (IRGs)^44^ and Atg5-dependent autophagy^45^. Finally, given that free fatty acids can have antibacterial activity^46^, AA might also directly affect chlamydial growth and development. Consistent with such a scenario, AA-treated chlamydial stocks display slightly lower infectivity than non-treated controls (Fig. 4K).

During maturation chlamydia-infected DCs display a pro-survival phenotype with increased autophagy, low ROS levels (Fig. 4G), and low numbers of apoptotic cells (Fig. 4H). This is in agreement with the idea that autophagy can suppress apoptosis in certain cellular contexts via degradation of defective mitochondria^47^. Harmful ROS production can rise when these organelles become dysfunctional^48^ and their removal through mitophagy prolongs cell survival by reducing mitochondrial apoptosis^49^. In DCs, chlamydia-infection as well as TNF-α or AA treatment strongly up-regulate glycolysis, which maintains ATP production despite the loss of functional mitochondria (Fig. 4I,J). Thus, infection drives a metabolic switch from oxidative phosphorylation to glycolysis, reminiscent of the Warburg effect^50^. Activation by pro-inflammatory stimuli causes a similar shift towards glycolysis and promotes survival^51^. Moreover, because loss of mitochondrial function correlates with reduced phago-/endocytic capacity^52^, metabolic re-programming probably also controls uptake and processing of antigens. Here, we demonstrate that this re-programming has additional critical effects on anti-bacterial immune defence (Fig. 4). The process allows infected DCs to restrict their infection by modulating the host environment. This program likely represents a critical feature of DC biology, allowing the uptake of infectious pathogens for antigen presentation while ensuring cellular survival and the suppression of microbial spread.

Chlamydiae exploit the host cell cytoskeleton to promote their survival and intracellular replication. They use MTs to migrate from the cell periphery towards the MTOC and Golgi^53^ and MT-dependent transport processes provide nutrients for the pathogen^54^. Consequently, disruption of chlamydial MTOC interaction drives bacterial inclusions into a non-productive functionally disordered state^55^. Moreover, chlamydial inclusions are enwrapped by acetylated MTs (Suppl. Fig. S4)^56^, which are normally found in stabilised MT structures in the MTOC/Golgi area^57^. HDAC6-mediated tubulin deacetylation promotes disassembly of this type of MTs^58^, but how cells modulate HDAC6 activity is poorly characterised. Recent studies suggest that TNF-α, which is also secreted by chlamydia-infected DCs^6^, induces HDAC6^59^. Conversely, HDAC6 inhibition affects TNF-α-triggered downstream effects^60^. Moreover, LPS also transiently regulates the expression of various HDACs including HDAC6 in APCs^61^. In agreement with these observations, we find that chlamydial infection of DCs strongly up-regulates HDAC6 expression (Suppl. Fig. S4). This dramatically alters the spatial distribution and abundance of acetylated MTs. In contrast to epithelial cells, chlamydial structures in DCs are not enwrapped by acetylated stable MTs and are spatially remote from the MTOC and the Golgi apparatus (Suppl. Fig. S4). Strikingly, HDAC6 inhibition in DCs fully rescues chlamydial compartments and mimics the phenotype observed in epithelial cells (Suppl. Fig. S4). This suggests that increased HDAC6 activity in DCs deprives chlamydia of acetylated MTs, which it needs for normal structural stabilization, growth, and development. Additionally, deacetylation of MTs also results in the collapse of vimentin^62^, another cytoskeletal protein required to protect the bacterial compartment^63^. That induction of cPLA2 (loss of mitochondrial function) and HDAC6 (deacetylation of stabilizing MTs) occurs within in the same time frame during infection strongly suggests that both mechanisms cooperate in the structural disintegration of chlamydial compartments. Thus, a coordinated immune defence downstream of TNF-α signalling appears to impair and destabilise the pathogen’s vacuoles and prime them for eventual destruction.

HDAC6 also seems to be a key factor promoting autophagic disposal of disintegrated chlamydial structures and decorates vimentin-containing aggresomes of mitochondrial and bacterial remnants (Figs 5, 8 and Suppl. Fig. S6). Its silencing reduces autophagy and suppresses bacterial antigen presentation (Figs 7, 8). Previous studies have reported a link between microtubule deacetylation and vimentin collapse, and HDAC6 activity appears to drive both^62^. Thus, a direct functional connection likely exists between the HDAC6-mediated MT destabilization and the intracellular formation of vimentin-containing aggregates that we observe in infected DCs (Figs 5, 6, 8 and Suppl. Fig. S4). Moreover, HDAC6 plays a critical role for autophagic aggresome clearance^64^. HDAC6 senses ubiquitinated aggregates and induces the expression of cellular chaperones such as HSP25/27^65^. HSP25/27 complexed with HDAC6, regulates MT architecture, modulates vimentin distribution and solubility^21^, and is associated with aggresomes^20^.

Damaged mitochondria are selectively degraded by mitophagy^66^. The process is initiated by the loss of Δψm, which stabilises Pink-1 on the outer mitochondrial membrane. Pink-1 acts as a sensor for damaged organelles and recruits the E3 ubiquitin ligase Parkin^67^. Recruitment of Parkin also requires the formation of mitochondrial aggregates that resemble aggresomes^68^. The accumulation of ubiquitinated mitochondrial proteins then causes the adapter p62 to bind, which in turn mediates interaction with LC3. Finally, LC3 facilitates the autophagosomal degradation of the damaged mitochondria^69^. Strikingly, we find that this entire complex of mitophagy machinery including Pink-1 (Figs 4B, 5C and Suppl. Fig. S2), Parkin (Fig. 7A,B and Suppl. Fig. S2), ubiquitin (Fig. 7A,B), p62 (Fig. 7A,B), and LC3 (Fig. 8A,B and Suppl. Fig. S2) undergoes coordinated targeting to disintegrated chlamydial structures. This strongly suggests that the resulting autophagy cascade plays a key role in the destruction of bacterial remnants together with their associated mitochondria and aggregates.

Autophagy also targets free bacteria in the cytosol, commonly referred to as xenophagy^5^. The process represents a defence mechanism against intracellular microbes including *Chlamydia spp* ^6, 70^. Mitophagy and xenophagy share critical molecular components including Parkin and p62^71^ and loss-of-function mutations in Parkin are associated with increased susceptibility to *Mycobacterium leprae* and *Salmonella enterica* ^72^. In a striking resemblance to mitophagy, Parkin was also recently found to mediate bacterial clearance^73^. Building on these results, we suggest that DCs eliminate intracellular chlamydia via a mito-xenophagic mechanism. In this process, mitochondria and structurally disintegrated inclusion remnants coaggregate into aggresomal structures. The Parkin/HDAC6/p62 pathway then transfers the aggregates together with the bacteria into autophagosomes for killing. Finally, chlamydial material accesses amphisomes, undergoes MHC class I processing, and is loaded onto recycling MHC I as described earlier^6^. In line with this, MHC I-mediated presentation of chlamydial antigens depends on TNF-α/p38/ERK1/2 signalling (Fig. 2I and Suppl. Fig. S3), cPLA2 activity (Fig. 3G), the mitophagy/aggrephagy-associated HDAC6/HSP25/27/Parkin machinery (Fig. 8E and Suppl. Fig. S7), and on autophagy factors like Beclin-1 and Atg7 (Fig. 1G and Suppl. Fig. S1).

**Figure 9:**
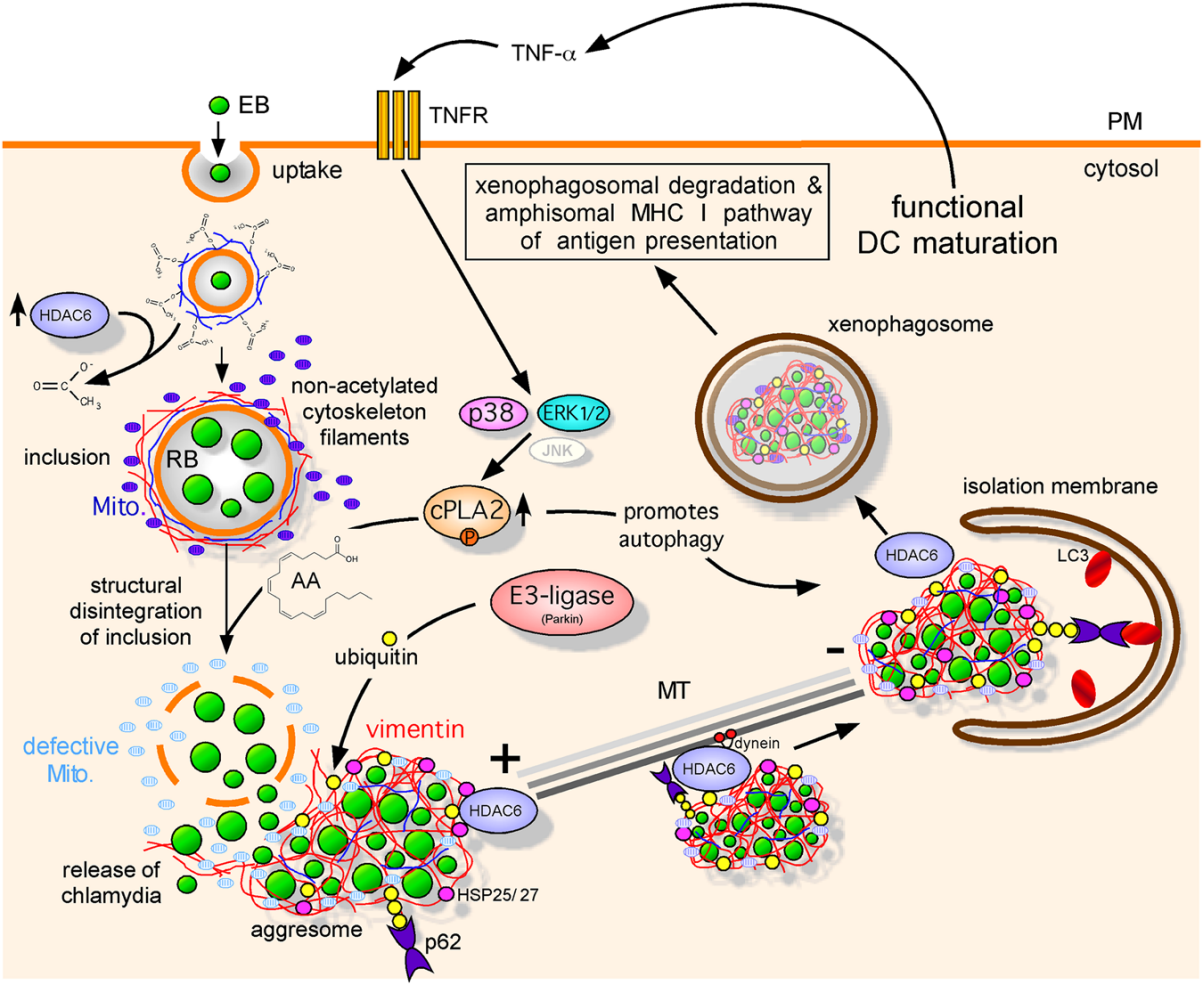
Model of mito-xenophagic degradation of bacterial structures in chlamydia-infected DCs. Infected DCs destroy inclusions resulting in the cytosolic release of bacteria. HDAC6-mediated deacetylation of stable MTs promotes inclusion disruption and a structural collapse of the associated vimentin. This is accompanied by a TNF-α mediated induction/activation of cPLA2, which catalyses the synthesis of AA. AA triggers disintegration of the fragile inclusions by affecting the proper function of inclusion-associated mitochondria. Since mitochondrial energy supply is essential for normal development and integrity of inclusions, a metabolic switch in infected DCs from OXPHOS to aerobic glycolysis causes irreversible damage to the bacteria. Chlamydial remnants are associated with collapsed vimentin, HDAC6, Parkin, small heat shock proteins (e.g. HSP25/27) as well as Pink-1-positive mito-aggresomes. Ubiquitinated aggresomal structures recruit p62, which binds to phagophore-conjugated LC3. Finally, xenophagosomes fuse with endo/lysosomal vacuoles. In parallel to the destruction of chlamydia, bacterial antigens are generated by the amphisomal pathway and are loaded onto MHC I derived from endosomal recycling. MHC I molecules successfully loaded with antigen are presented on the cell surface to CD8^+^ T cells.

Based on our present findings, we propose the following model (Fig. 9): Chlamydiae surround their vacuole with a mesh of cytoskeletal filaments (e.g. MTs) to structurally support their compartment. DCs use HDAC6 to break down this stabilizing scaffold, and thereby promote the disruption of bacterial vacuoles^6^. This is accompanied by TNF-α-mediated induction and phospho-activation of cPLA2. Activated cPLA2 produces AA, which then destabilises the inclusions further by inducing a loss of Δψm in associated mitochondria^74^. The concomitant metabolic switch from oxidative phosphorylation to aerobic glycolysis cuts the essential delivery of energy and nutrients to the pathogen. Following the disintegration of inclusions, released cytosolic bacteria coaggregate with defective mitochondria, which recruit the HDAC6/HSP25/27/Parkin machinery. The mito-aggresomal structures undergo ubiquitination and recruit LC3 via the adaptor protein p62. The resulting xenophagosomes eventually fuse with endo/lysosomal vacuoles to generate amphisomes and autolysosomes, which drive subsequent MHC I antigen presentation^6^.

## Methods

### Cell culture

JAWSII, a murine (C57BL/6) myeloid DC line established from bone marrow (BM) cells^7^, was purchased from ATCC (CRL-11904). Immortalised epithelial cells from new-born mice were obtained from the Collection of Cell Lines in Veterinary Medicine (CCLV) of the Friedrich-Loeffler-Institut (CCLV-RIE #282)^6^. The epithelial African green monkey kidney cell line BGM^75^ was obtained from the National Reference Laboratory for Chlamydiosis of the Friedrich-Loeffler-Institut (CCLV-RIE #136). Primary BMDCs (C57BL/6) were produced after 7–14 days of bone marrow cells culture in GM-CSF (5–10 ng/ml)-containing medium (IMDM) as described by Winzler *et al*.^76^ and assessed for purity by flow cytometry (CD11c, CD40, CD86, CD80 and MHC I/II staining). Cells were grown at 37 °C and 7.5% CO_2_ in IMDM/5–20% FCS. Murine GM-CSF at 5–10 ng/ml was used for culturing DCs.

### Antibodies

Antibodies against chlamydia, chlamydial LPS, GAPDH, Atg7, Parkin, HDAC6, cPLA2 (phosphorylated and non-phosphorylated), H-2K^b^, H-2D^b^, CD80, CD86, PD-L1, PD-L2, p38, P-p38, ERK1/2, P-ERK1/2, MAPKAPK-2, P-MAPKAPK-2, JNK, P-JNK, cJun, P-cJun, HSP25/27, P-HSP25/27, vimentin, Pink-1, p62, ubiquitin, acetylated α-tubulin, tubulin and β-actin were obtained from Sigma-Aldrich, Progen, Abcam, Upstate, eBioscience, CellSignaling, Thermo Scientific Pierce, antibodies-online and Millipore, respectively. Anti-Beclin-1, anti-LC3, and anti-chlamydial HSP60 were purchased from Abcam, Abgent Europe and Acris, respectively. Secondary and isotype-control antibodies were purchased from Dianova and BioLegend.

### Western blotting

Cells were lysed on ice in RIPA buffer (150 mM NaCl, 50 mM Tris-HCl, 1% NP-40, 0.25% Na-deoxycholate, and cOmplete protease inhibitor (Roche), 50 mM NaF) with 4 M urea. Postnuclear supernatants were analysed in Western blot as described^6^. Fluorographs were quantified with GelEval 1.32 (FrogDance Software).

### Chlamydia

The non-avian *C. psittaci* strain DC15^8^ was grown in BGM cells. Chlamydial EBs were purified by discontinuous density-gradient ultracentrifugation^77^ using Visipaque (Nycomed). Purified EBs were stored in sucrose-phosphate-glutamic acid buffer at −80 °C. Infection forming units (IFUs) were determined by immunostaining (IMAGEN kit, Oxoid). Unless indicated otherwise, cells were infected with EBs at an MOI of 5–10.

### Flow cytometry, microscopy, and colorimetry

Flow cytometry was performed as described previously^6^. Cells were analysed on a MACSQuant analyser (Miltenyi Biotec). Viability was assessed with trypan blue. For chlamydial staining and titer determination, cells were fixed with 2% paraformaldehyde, permeabilized in PBS/0.5% saponin/0.5% BSA at RT for 30 min and immunostained with the IMAGEN kit (Oxoid). Immunofluorescence microscopy was performed as described^6^. Autophagosome and aggresome formation were analysed using the CytoID (with a cationic amphiphilic tracer (CAT)) as fluorescence reagent) and Proteostat kits (Enzo Life Sciences). Rapamycin was used as autophagy inducer. Mitochondrial membrane potential (MMP, Δψm) was measured with the JC-1 kit (Abcam). As a control, the mitochondrial oxidative phosphorylation (OXPHOS) uncoupler 2-[2-(3-Chlorophenyl) hydrazinylyidene] propanedinitrile (CCCP) was used at 50 µM. Reactive oxygen species (ROS) production was analysed by cellROX green staining (Life Technologies). 2-Hydroperoxy-2-methylpropane (TBHP, 200 μM) was used as control reagent for cellROX green staining. ATP was measured using the ATP Assay kit (Abcam). For the detection of apoptotic and necrotic cells the Annexin V-FITC kit (Miltenyi) was used. Cellular lactate was determined with L-Lactate Assay Kit (Abcam).

### DC/T cell cocultivation assay and IFN-γ ELISA

1 × 10^5^ DCs (JAWSII) were incubated with EBs for 24 h. Infected DCs were cocultured with 5 × 10^5^ chlamydia-sensitised CD8^+^ T cells from *C. psittaci*-infected mice in 200 μl fresh medium for 48 h. Culture supernatants were collected and IFN-γ was assayed by Mouse IFN-γ Platinum ELISA (eBioscience) on a Microplate ELISA Reader (Sunrise Remote, Tecan) at 405 nm. Chlamydia-specific CD8^+^ T cells were generated by immunizing mice i.p. with 5 × 10^7^ IFU live EBs and boosting 2 and 3 weeks later as previously described^9^. After 4 weeks, mice were challenged intranasally with 5 × 10^5^ IFU. T cells were purified from murine spleens by negative selection using the CD8^+^ T Cell Isolation Kit II (Miltenyi Biotec). As a control, CD8^+^ T cells from naive mice were purified. CD8^+^ T cells with ≥90% purity were obtained, as measured by flow cytometry. CD8^+^ T cells, cultured with anti-CD3/CD28-conjugated beads (Dynabeads, mouse, Invitrogen) were used as a positive control. All animal procedures were approved by the local District Government (State Office for Agriculture, Food Safety and Fishery in Mecklenburg-Western Pomerania - LALFF M-V), (registration number 7221.3-1.1-047/11) and were carried out in accordance with the relevant guidelines and regulations of the German law for the protection of animal life.

### siRNA-mediated gene silencing

4 × 5 μg oligo duplex RNAs specific for Beclin-1 (5′-CGGGAGTATAGTGAGTTTAAA-3′, 5′-TTGGGTAATATTAAACCACAT-3′, 5′-TTGGTTTGGAAAGATGCTTTA-3′, 5′-CGGACA GTTTGGCACAATCAA-3′); Atg7(5′-CAGCCTCTGTATGAATTTGAA-3′, 5′-AAGGTCAAAG GACAAAGATAA-3′, 5′-TAGCATCATCTTTGAAGTGAA-3′, 5′-CAGCTCTGAACTCAATAAT AA-3′); cPLA2 (5′-CTGAACAACATTGATGTGATA-3′, 5′-TAGGAGAAACACTAATTCAAA-3′, 5′-AAGCCTGAGGATTCTCATTTA-3′, 5′-CCAGATGAATTTGAACGAATA-3′); Parkin (5′-CACTGTGAATTTAACAGAGAA-3′, 5′-CACCAGCATCTTGCAGCTCAA-3′, 5′-CAGGGAG GACTCAGAAGCCAA-3′, 5′-CTGGAACAACAGAGTATTGTA-3′); HSP25/27 (5′-TCCGGAG GAGCTCACAGTGAA-3′, 5′-CACAGCCGCCTCTTCGATCAA-3′, 5′-TCGGTGCTTCACC CGGAAATA-3′, 5′-GCGCGTGTCCCTGGACGTCAA-3′); and HDAC6 (5-TTGGTGTTTG ATGAACAACTA-3′, 5′-CAGGTGCTTATTTAAGTACAA-3′, 5′-GAGGATGACCCTAGTGTA TTA-3′, 5′-CCGGCCAAGATTCTTCTACTA-3′) were purchased from QIAGEN and transfected into 5 × 10^5^ cells using the nucleofector system and the Mouse Dendritic Cell Nucleofector kit (Lonza). As a negative control, AllStars siRNA (QIAGEN) was used. Cells were maintained for up to 48 h and screened for gene silencing by Western blot.

### RT-PCR

Total RNA from DCs was isolated and analysed by semi-quantitative RT-PCR for HSP25/27, chlamydial (chl.) HSP60 and GAPDH. The respective PCR primer pairs were: 5′-ACAGCTCAGCAGCGGGGTCT-3′, 5′-GGCGCGGGCCTCGAAAGTAA-3′ (mouse HSP25/27); 5′-CACCTTCGATGCCGGGGCTG-3′, 5′-TGTTGGGGGCCGAGTTGGGA-3′ (mouse GAPDH); 5′-CAACAGGTAGCAGAATCCGGA-3′, 5′-CTCTTCGCTGATAAGTTGG CCA-3′ (chl.HSP60 (groEL)).

### Transmission electron microscopy

DCs were fixed for 2 h at 4 °C with 2.5% glutaraldehyde in cacodylate buffer (0.1 M, pH 7.2) 48 h post infection and centrifuged for 5 min at 1500 × g. The pellet was embedded in 2% agarose and sectioned into 1 mm^3^ cubes. Cubes were post-fixed in 2% osmium tetroxide and embedded in Araldite Cy212. Ultrathin sections (85 nm) were stained with uranyl acetate and lead citrate and examined by transmission electron microscopy (TEM, Tecnai 12, FEI)^6^.

### Aggresome purification and mass spectrometry (MS) analysis of aggresomes

Both experimental procedures are described in the supplementary methods.

### Statistical analysis

Statistical analysis of the obtained data is shown as the mean ± SD of three individual experiments and was estimated using GraphPad Prism 6 (GraphPad Software). Data were analysed by t-test and one-way analysis of variance (ANOVA) followed with Dunnett’s and/or Tukey’s post hoc test (n.s.: not significant; *p < 0.05; **p < 0.01 and ***p < 0.001).

### Data availability

All data generated or analysed during this study are included in this published article (and its Supplementary Information files).

## Electronic supplementary material

Supplementary information
