# Supplementary material for: Mito-xenophagic killing of bacteria is coordinated by a metabolic switch in dendritic cells
Source: Sci Rep. 2017 Jun 20;7:3923. doi: 10.1038/s41598-017-04142-5 (PMC5478671; doi:10.1038/s41598-017-04142-5)
Supplement: Supplementary file 1 — Supplementary information [file 41598_2017_4142_MOESM1_ESM.pdf]

Supplementary Information

Mito-xenophagic killing of bacteria is coordinated by a metabolic switch in dendritic cells

Nadine Radomski[a], Danny Kägebein[b], Elisabeth Liebler-Tenorio[c], Axel Karger[d], Elke Rufer[a], Birke Andrea Tews[d], Stefanie Nagel[b], Rebekka Einenkel[a], Anne Müller[e], Annica Rebbig[a], and Michael R. Knittler[a*]

[a]Institute of Immunology, Friedrich-Loeffler-Institut,
Federal Research Institute of Animal Health,
Südufer 10, D-17493 Greifswald, Isle of Riems, Germany

[b]German Cancer Research Center (DKFZ)
Im Neuenheimer Feld 280
D-69120 Heidelberg, Germany

[c]Institute of Molecular Pathogenesis, Friedrich-Loeffler-Institut,
Federal Research Institute of Animal Health,
Naumburger Strasse 96a, D-07743 Jena, Germany

[d]Institute of Molecular Virology and Cell Biology, Friedrich-Loeffler-Institut,
Federal Research Institute of Animal Health,
Südufer 10, D-17493 Greifswald, Isle of Riems, Germany

[e]University of Tübingen, Interfaculty Institute of Biochemistry
Hoppe-Seyler-Straße 4, D-72076 Tübingen, Germany

[*]**Corresponding author:**
Dr. Michael R. Knittler
Institute of Immunology,
Friedrich-Loeffler-Institut
Federal Research Institute of Animal Health
Südufer 10
D-17493 Greifswald-Insel Riems/ Germany
Phone: +49 383517-1170
Fax: +49 383517-1643
e-mail: michael.knittler@fli.de

Supplementary Methods

Aggresome purification

Aggresomal structures were isolated as described [1]. Cells were scraped into 400 µl PBS/Complete Protease Inhibitor (Roche), Dounce-homogenised (30 strokes / tight pestle), and incubated for 60 min at 37°C in the presence of 10 mM $MgCl_2$ and 40 U/ml DNase A (Roche). A sucrose step gradient in PBS was prepared (2 ml 80%, 2 ml 50%, 2 ml 20%) and overlaid with the cell homogenate (1 ml) prior to centrifugation at 1000 xg (15 min/4°C). The aggresome-containing interphase between the 50-80% sucrose was collected, mixed with 5 volumes of PBS, and again centrifuged at 1000 xg (15 min/4°C). The supernatant was discarded and the aggresome pellet was suspended in 250-500 µl PBS.

MS analysis of aggresomes

Isolated vacuum-dried aggresomes were extracted with 8 M urea/NH_4HCO_3 via sonication. DTT-reduced samples were alkylated with iodoacetamide and trypsin-digested. Peptides were recovered by solid phase extraction and dissolved in 0.1% trifluoroacetic acid for nano liquid chromatography (nLC). Peptides were analysed by nLC MALDI-TOF/TOF MS on a platform consisting of nLC and UltrafleXtreme MALDI-TOF/TOF (Bruker). Fractionation was carried out on an Acclaim PepMap100 column (Thermo Scientific) with an acetonitrile gradient (2-45%). Mass spectrometric analysis was launched by WARP-LC software (Bruker) and the fragment spectra were passed to a MASCOT server. The Swissprot [2] database was used and restricted to *Mus musculus* entries for the query of the host proteins. For chlamydial proteins, a database was set up using the sequences of *C. psittaci* 02DC15 (Genbank NC_017292.1, [3]). MASCOT results were exported to ProteinScape software (Bruker) for further evaluation. Protein identities were reconstructed from the peptide spectra by the Protein Extractor (PE) feature of ProteinScape. The minimum MASCOT peptide score was set to 15 and the significance level to 0.95. Proteins were quantitated on the basis of the exponentially modified protein abundance index (emPAI) calculated according to Ishihama et al. [4].

---

1 Bader, V., Ottis, P., Pum, M., Huston, J. P. & Korth, C. Generation, purification, and characterization of cell-invasive DISC1 protein species. *J. Vis. Exp.* **66**, e4132 (2012).
2 Magrane, M. UniProt Knowledgebase: a hub of integrated protein data. *Database (Oxford)* **2011**, bar009 (2011).
3 Schöfl, G., Voigt, A., Litsche, K., Sachse, K. & Saluz, H. P. Complete genome sequences of four mammalian isolates of *Chlamydophila psittaci*. *J. Bacteriol.* **193**, 4258 (2011).
4 Ishihama, Y. *et al.* Exponentially modified protein abundance index (emPAI) for estimation of absolute protein amount in proteomics by the number of sequenced peptides per protein. *Mol. Cell. Proteomics* **4**, 1265-1272 (2005).

Supplementary Figures

Fig. S1 siRNA silencing of Beclin-1 or Atg7 has no detectable effect on expression or infection-induced increase of surface MHC I and coregulatory molecules (CD80, CD86, PD-L1 and PD-L2)

Fig. S2 Chlamydia-infected primary BMDCs display disintegration of inclusions and colocalisation between autophagic factors and bacterial structures

Fig. S3 Role of MAPKs ERK1/2, p38 and JNK in cPLA2-activation of chlamydia-infected DCs

Fig. S4 Infected DCs are characterised by chlamydial structures lacking acetylated α-tubulin filaments

Fig. S5 TNF-α is involved in the phosphorylation but not in the upregulation of HSP25/27 in chlamydia-infected DCs

Fig. S6 Aggresomes of infected DCs are characterised by chaperones, HDAC6, cytoskeletal, mitochondrial and chlamydial proteins

Fig. S7 Parkin/HSP25/27/HDAC6-triple-silencing blocks infection-induced autophagy, shows a striking increase in the bacterial load and strongly affects chlamydial antigen presentation in infected DCs

Fig. S1

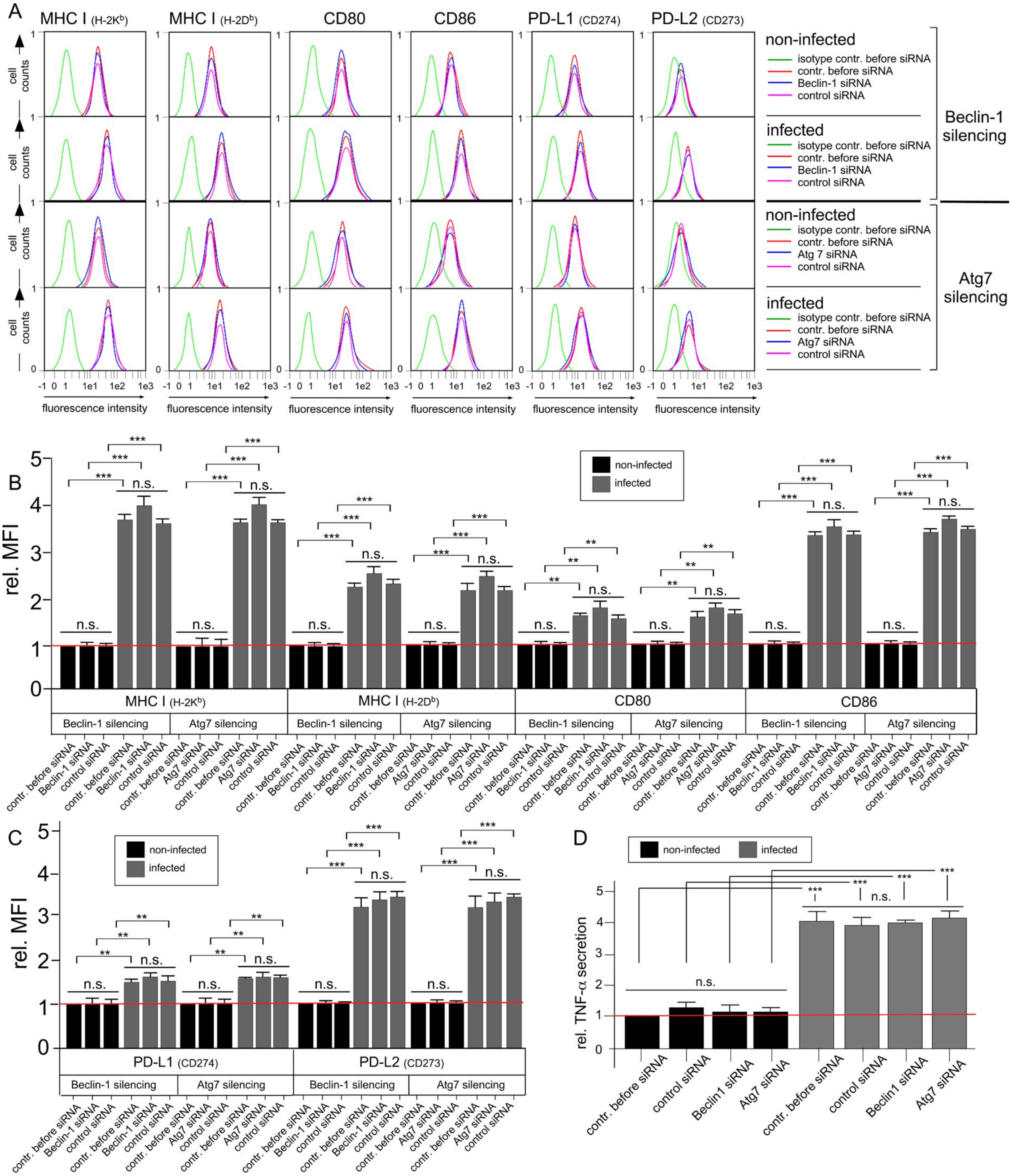

Fig. S1 siRNA silencing of Beclin-1 or Atg7 has no detectable effect on expression or infection-induced increase of surface MHC I and coregulatory molecules (CD80, CD86, PD-L1 and PD-L2). A) To investigate surface expression of MHC I and coregulatory molecules before and after knockdown of Beclin-1 and Atg7 in response to infection, DCs were siRNA-silenced or not and then infected with chlamydia (48 hpi). AllStars siRNA (control siRNA) and non-infected cells were used in control experiments. Samples (before/after specific silencing and control siRNA treated cells) were analysed by flow cytometry using antibodies specific for MHC I (H-$2K^b$, H-$2D^b$), coregulatory molecules CD80, CD86, PD-L1 and PD-L2 as well as respective isotype controls. One representative isotype control (non-infected cells before siRNA) is shown for all groups for simplicity as individual controls were similar between conditions. **B, C)** Results of the flow cytometry experiments are summarised as bar graphs with arbitrary units. MFIs obtained for non-infected cells before knockdown were set to 1 arbitrary unit (indicated by red line). All data are represented as the means ± SD from three independent experiments. Statistical analysis was performed as described in Methods (n.s.: not significant; **$p < 0.01$; ***$p < 0.001$; n=3). **D)** DCs were siRNA-silenced or not and then infected with chlamydia (48 hpi). AllStars siRNA (control siRNA) and non-infected cells were used in control experiments. TNF-α secreted by cultured DCs (before/after specific silencing and control siRNA treated cells) was assayed by TNF-α ELISA (R&D Systems). Relative values for TNF-α secretion are expressed in arbitrary units (values obtained for controls before silencing were set to 1) and are means ± SD of three independent experiments. Statistical analysis was performed as described in Methods (n.s.: not significant; ***$p < 0.001$; n=3).

Fig. S2

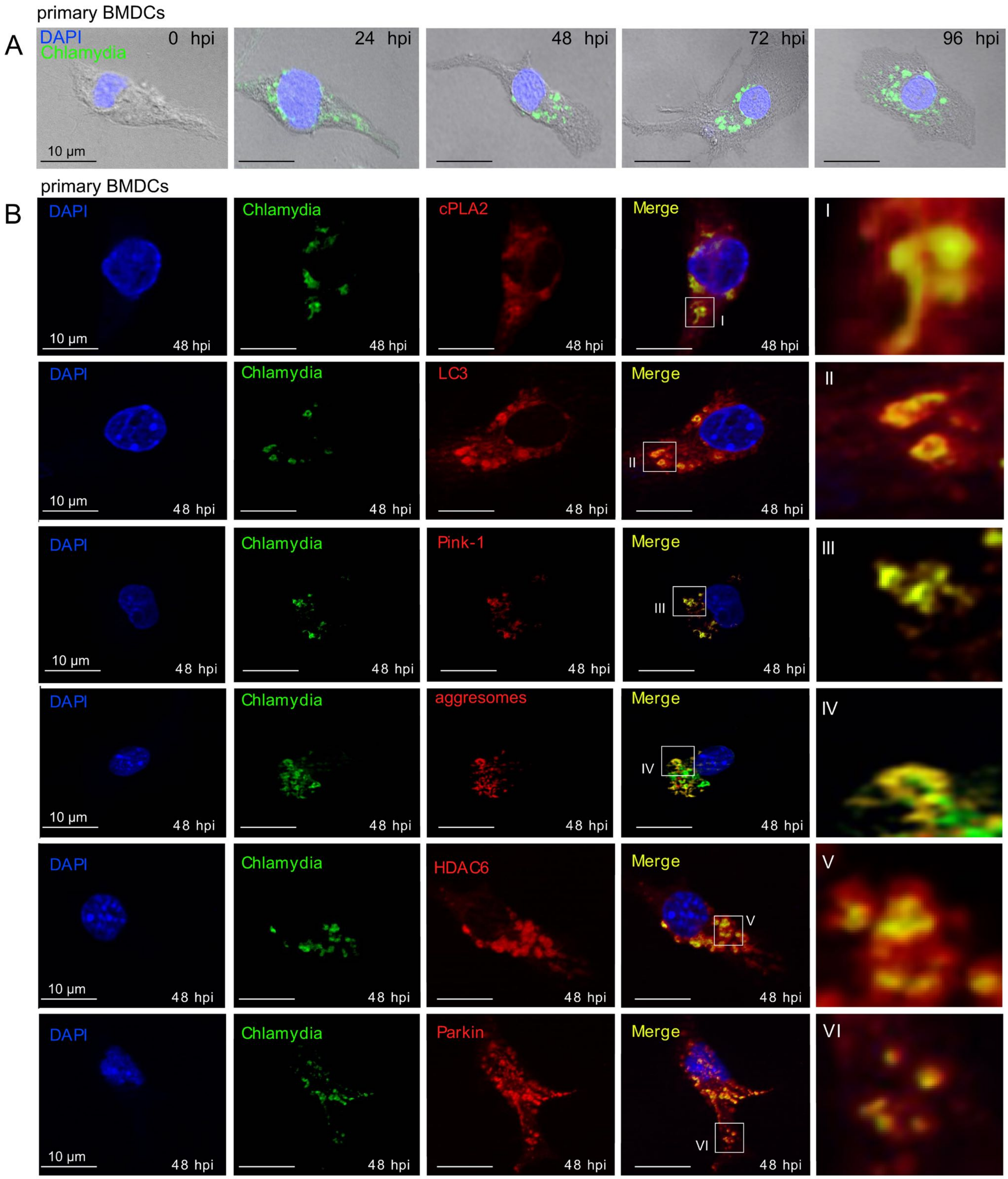

Fig. S2 Chlamydia-infected primary BMDCs display disintegration of inclusions and colocalisation between autophagic factors and bacterial structures. A) Infected primary BMDCs (0, 24, 48, 72 and 96 hpi) were stained for intracellular chlamydial structures (green) and DNA (blue). **B)** Primary BMDCs were infected with chlamydia (48 hpi) and costained for cPLA2, LC3, Pink-1, aggresomes, HDAC6, Parkin (red), and chlamydial structures (green). DNA was visualised by DAPI (blue). Enlarged photographs (I-VI) show colocalisation between cellular proteins/structures and chlamydia.

Fig. S3

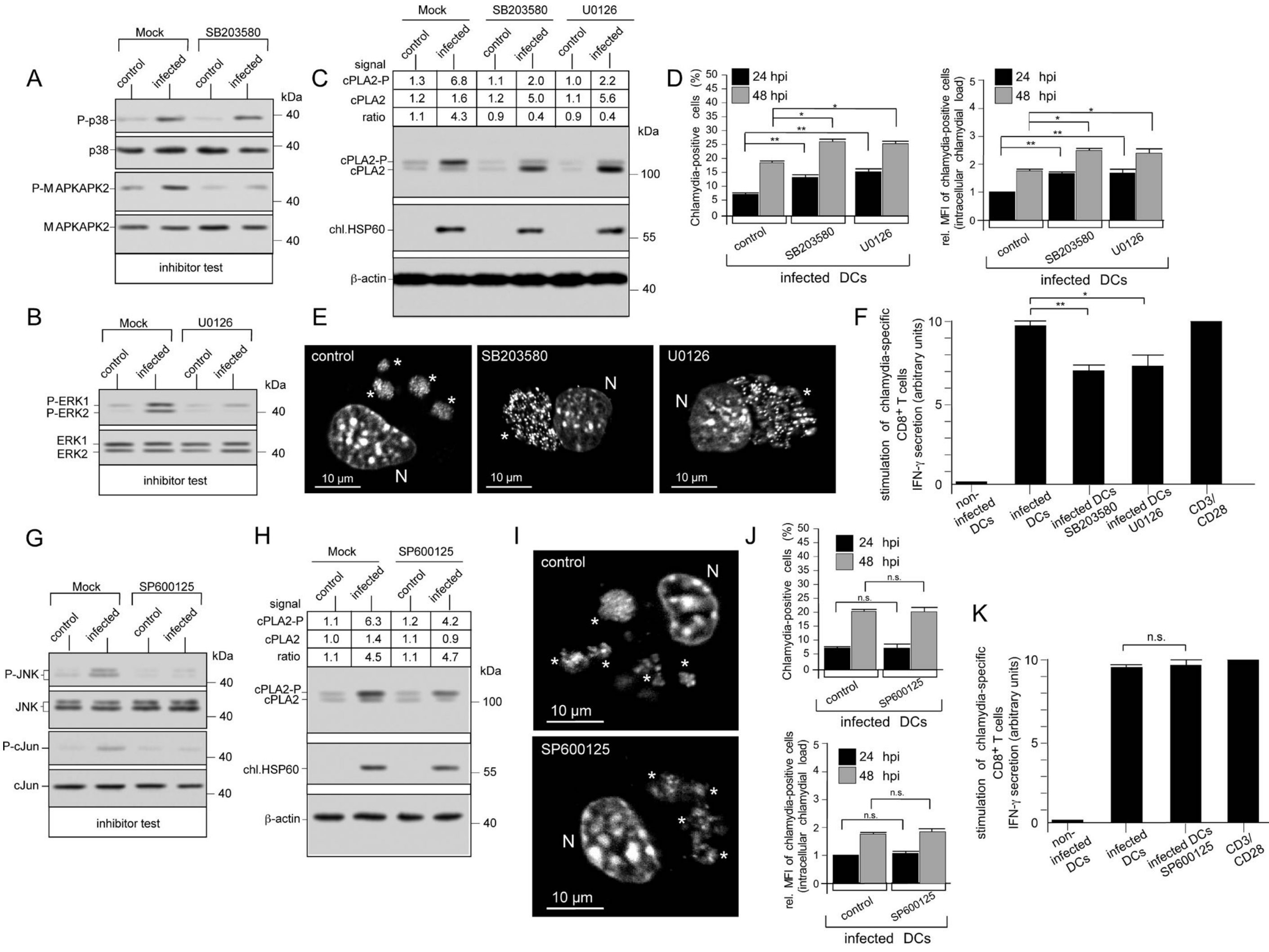

Fig. S3 Role of MAPKs ERK1/2, p38 and JNK in cPLA2-activation of chlamydia-infected DCs. A) Inhibitor test for SB203580**.** Western blots of infected (12 hpi) and non-infected DCs treated or not with p38 inhibitor SB203580 (10 µM) using p38, P-p38, MAPKAPK2 and P-MAPKAPK2 reagents. **B)** Inhibitor test for U0126. Western blots of infected (12 hpi) and non-infected DCs treated or not with MEK inhibitor U0126 (25 µM) using ERK1/2, P-ERK1/2 reagents. **C)** Western blot of infected DCs (24 hpi) treated with SB203580 or U0126 using antibodies against cPLA2, chl.HSP60, and β-actin. cPLA2-P and cPLA2 levels (and ratios) were determined by densitometric scanning. **D)** Flow cytometry of inhibitor (SB203580 or U0126) treated cells using the IMAGEN kit. Data from three independent experiments (24 and 48 hpi) are summarised in bar graphs. Statistical analysis was performed as described in Methods (*p < 0.05; **p < 0.01; n=3). **E)** Microscopy of DAPI-stained infected DCs (48 hpi) treated with SB203580 or U0126. **F)** IFN-γ secretion by chlamydia-specific CD8$^+$ T cells stimulated with infected DCs treated with SB203580 or U0126. Relative values of IFN-γ secretion are expressed in arbitrary units (maximum value was set to 10) and are means ± SD of three independent experiments. Statistical analysis was performed as described above (*p < 0.05; **p < 0.01; n=3)**. G)** Inhibitor test for SP600125. Western blots of infected (12 hpi) and non-infected DCs treated or not with JNK inhibitor SP600125 (25 µM) using JNK, P-JNK, cJun and P-cJun reagents. **H)** Western blot of infected DCs (24 hpi) treated with SP600125 using antibodies against cPLA2, chl.HSP60, and β-actin. cPLA2-P and cPLA2 levels (and ratios) were determined by densitometric scanning. **I)** Microscopy of DAPI-stained infected DCs (48 hpi) treated with SP600125. **J)** Flow cytometry of inhibitor (SP600125) treated cells using the IMAGEN kit. Data from three independent experiments (24 and 48 hpi) are summarised in bar graphs. Statistical analysis was performed as described above (n.s.: not significant; n=3). **K)** IFN-γ secretion by chlamydia-specific CD8$^+$ T cells stimulated with infected DCs treated with SP600125. Relative values of IFN-γ secretion are expressed in arbitrary units (maximum value was set to 10) and are means ± SD of three independent experiments. Statistical analysis was performed as described in Methods (n.s.: not significant; n=3)**.**

Fig. S4

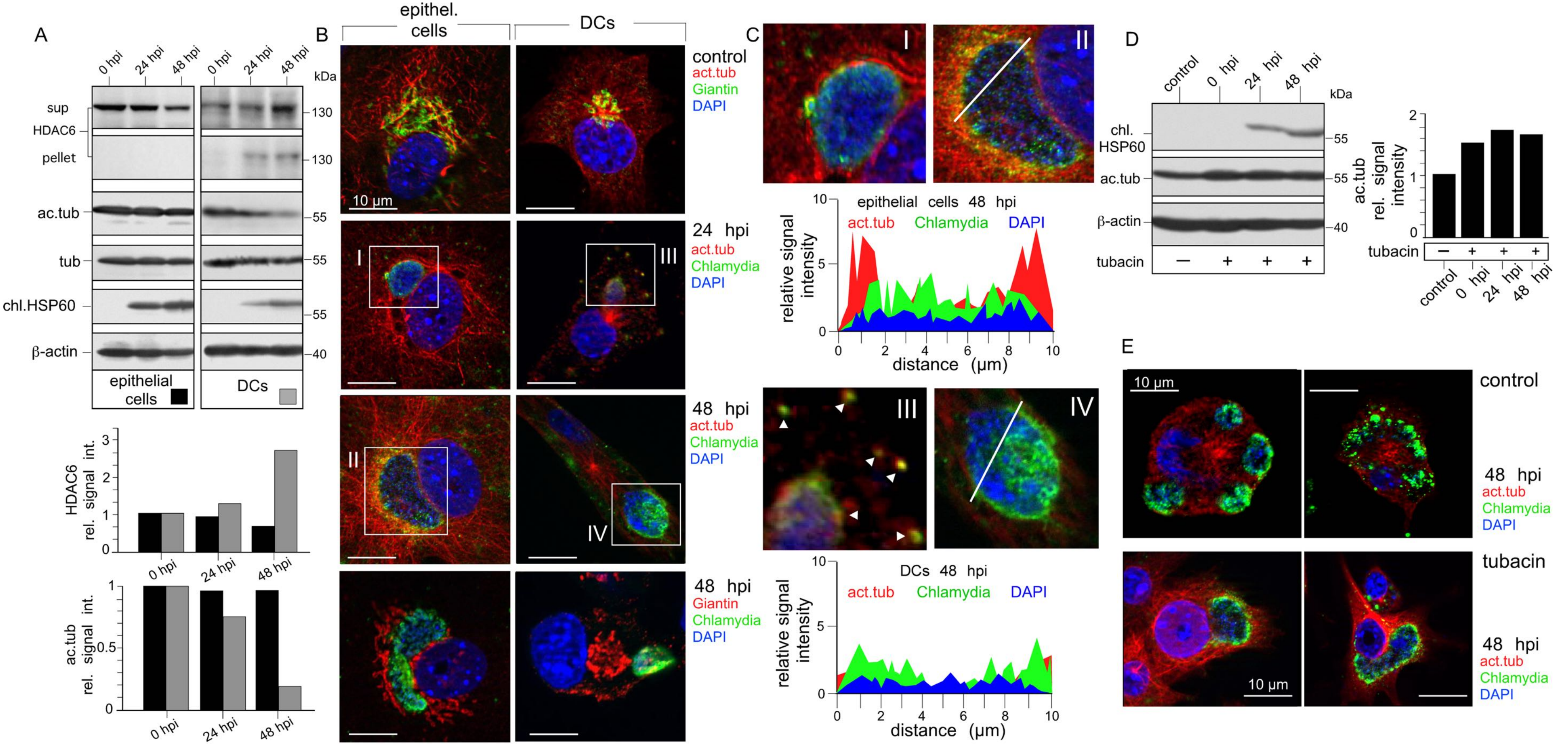

Fig. S4 Infected DCs are characterised by chlamydial structures lacking acetylated α-tubulin filaments. A) DCs and epithelial cells were infected or not with chlamydia. After cell lysis, HDAC6 (supernatant and pellet fraction), acetylated α-tubulin (ac.tub), total α-tubulin (tub) and chl.HSP60 were detected by Western blot (upper panel). HDAC6 (soluble fraction) and acetylated α-tubulin were quantified by densitometric scanning (lower panel). After normalization for β-actin, signals of HDAC6 and acetylated α-tubulin in non-infected control cells (0 hpi) were set to 1 arbitrary unit. **B)** Non-infected and infected (24 and 48 hpi) DCs and epithelial cells were costained for acetylated α-tubulin (red), Giantin (red or green), chlamydia (green) and DNA (blue). **C)** Enlarged images (I, II, III and IV of **Fig. B**) display chlamydial structures and distribution of acetylated α-tubulin (first and third row from the top). Chlamydial structures are indicated by arrow heads. The fluorescence intensity along a cellular cross section of interest was measured (second and fourth row from the top). Obtained profiles were overlaid and coloured. **D)** Tubacin treated DCs were infected or not with chlamydia. After cell lysis, chl.HSP60, acetylated α-tubulin and β-actin were detected by Western blot (left panel). Measured signals of acetylated α-tubulin were normalised for β-actin and plotted in a respective graph as arbitrary units (right panel). **E)** Infected DCs treated or not with tubacin were costained for acetylated α-tubulin (red), chlamydia (green) and DNA (blue).

Fig. S5

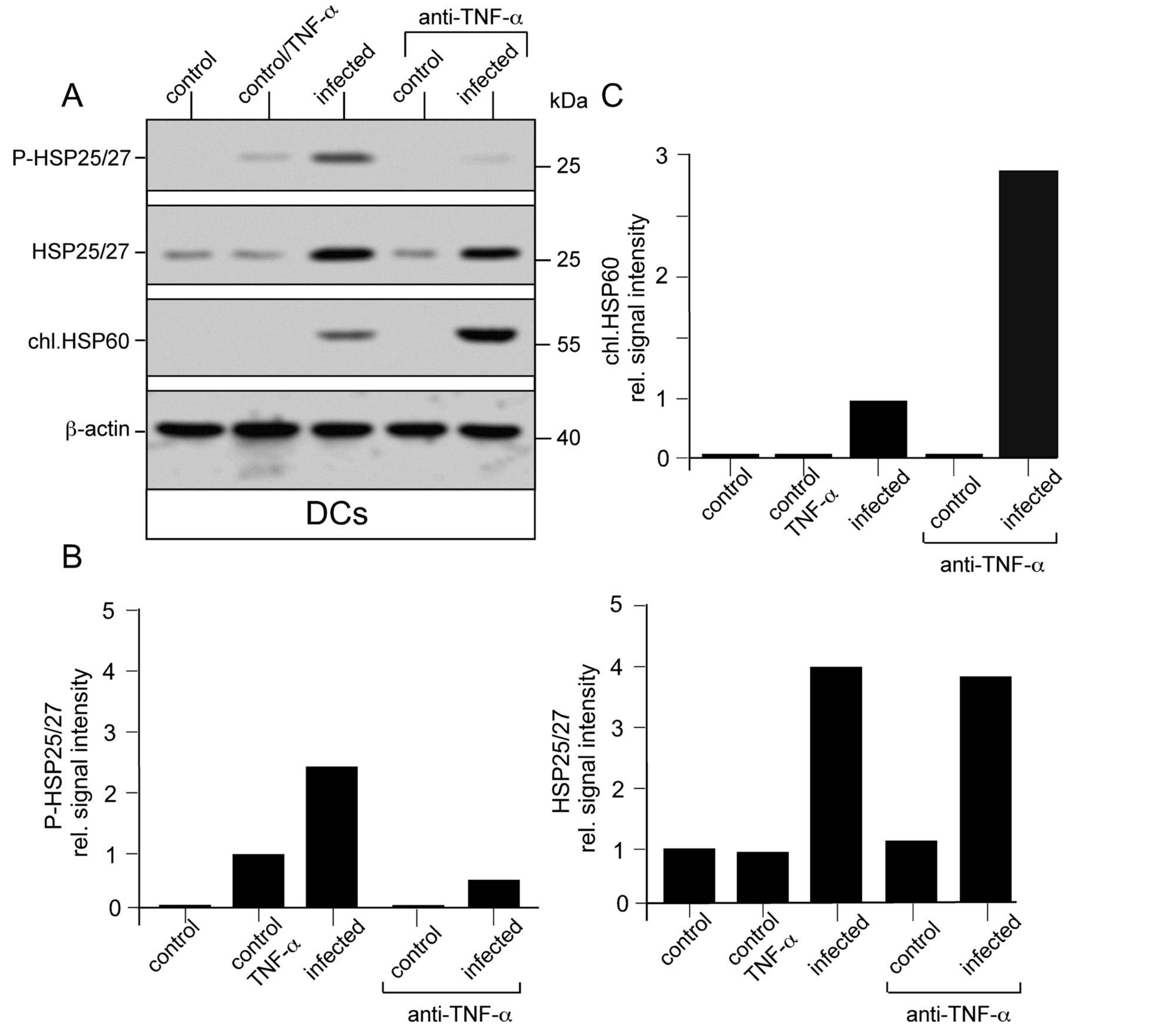

Fig. S5 TNF-α is involved in the phosphorylation but not in the upregulation of HSP25/27 in chlamydia-infected DCs. A) Western blots of infected and non-infected DCs treated or not with TNF-α-neutralizing antibodies using P-HSP25/27 (phosphorylated chaperones), HSP25/27 (all chaperones) , chl.HSP60, and β-actin-specific reagents. TNF-α treated non-infected DCs were used as additional controls. P-HSP25/27 (**B**, left panel), HSP25/27 (**B**, right panel) and chl.HSP60 **(C)** signals were determined by densitometric scanning, normalized for β-actin and plotted in respective graphs as arbitrary units. Signal values obtained for treated control (**B**, left panel, TNF-α treated control, P-HSP25/27 stained), untreated control (**B**, right panel, control, HSP25/27 stained) or infected cells (**C**, infected, chl.HSP60 stained) were set to 1.

Fig. S6

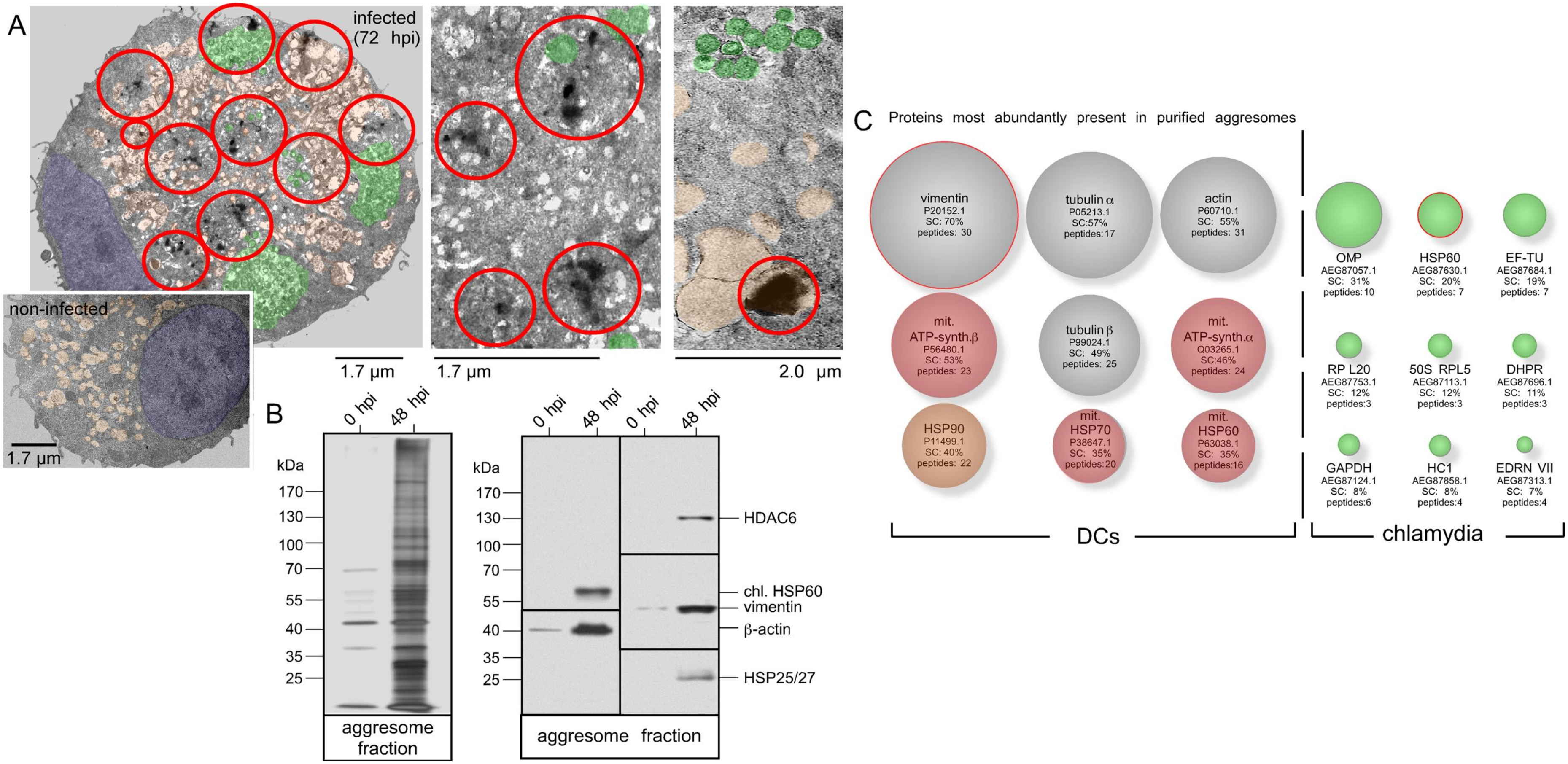

Fig. S6 Aggresomes of infected DCs are characterised by chaperones, HDAC6, cytoskeletal, mitochondrial and chlamydial proteins. A) Electron photomicrographs of non-infected (lower left panel) and infected DCs (72 hpi; upper left panel). The magnification (middle panel) shows aggregated electron dense material (red circles) in close vicinity to inclusions and released cytosolic chlamydial structures (artificially coloured in green). Some infected DCs showed also electron dense structures inside intact vacuoles (right panel). Nucleus and vacuolar structures are artificially coloured in blue and orange, respectively **B)** Isolated aggresomal structures from infected and non-infected DCs were separated by SDS-PAGE (silver stained, left panel) and analysed by Western blot (right panel) for the presence of chl.HSP60, HDAC6, vimentin, β-actin and HSP25/27. **C)** Aggresomes from infected DC cultures were further analysed by nLC MALDI-TOF/TOF MS. Among the most abundant proteins typical aggresome constituents were identified. In addition, several chlamydial proteins were found. The figure shows an overview of these highly abundant cellular (left panel) and bacterial proteins (right panel). Each of the identified proteins is symbolised by a circle with a defined area, which directly corresponds to the respective sequence coverage (in percent). chl.HSP60 and vimentin were used as host and bacterial reference proteins (identified in Western blots) and are indicated by a red border.

Fig. S7

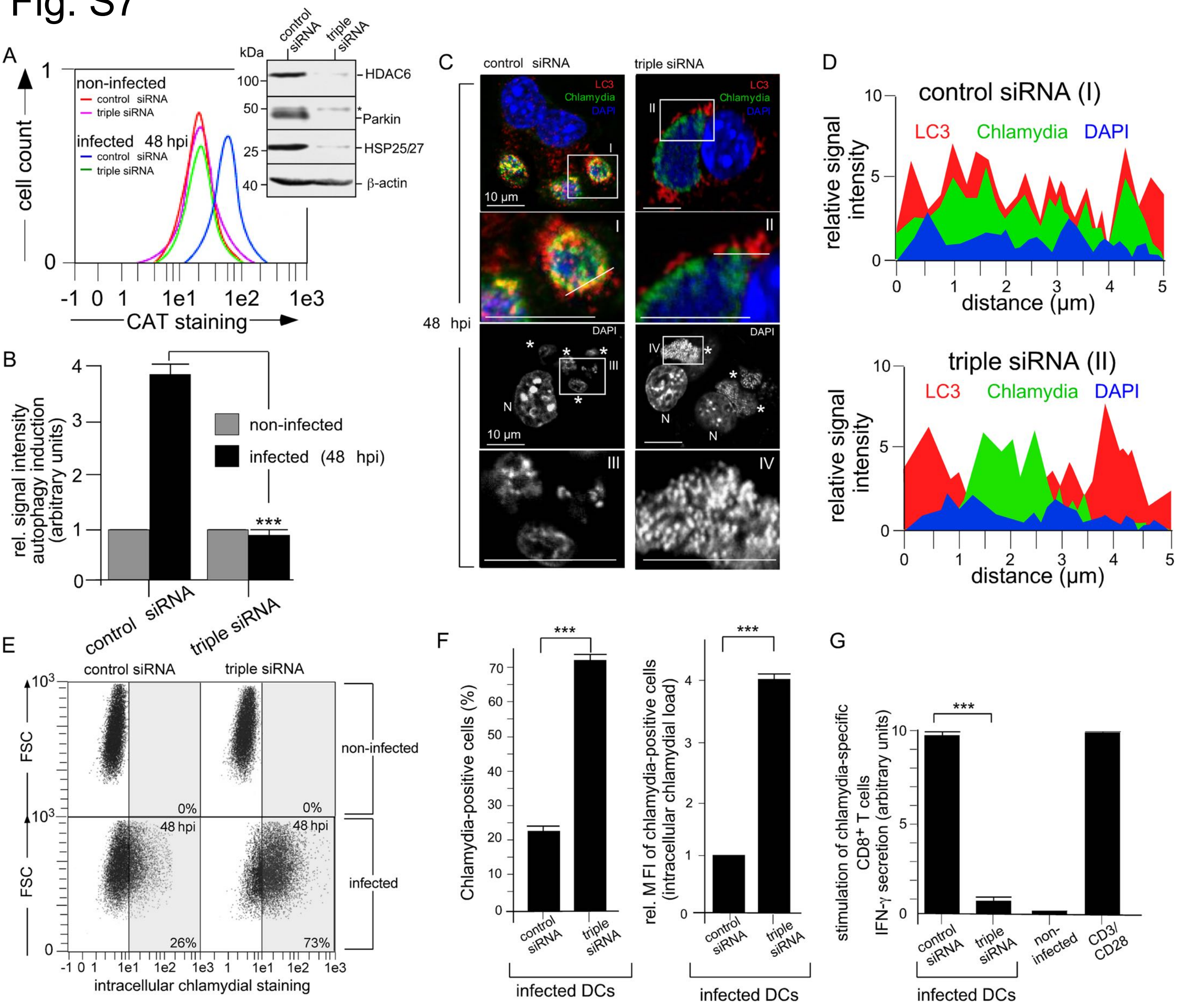

Fig. S7 Parkin/HSP25/27/HDAC6-triple-silencing blocks infection-induced autophagy, shows a striking increase in the bacterial load and strongly affects chlamydial antigen presentation in infected DCs. A triple combination of siRNAs simultaneously targeting Parkin, HSP25/27 and HDAC6 was used to knockdown all three aggrephagy-associated proteins in chlamydia infected DCs. AllStars siRNA was used as a control. **A)** Triple-silencing of Parkin/HSP25/27/HDAC6 was demonstrated by Western blot (upper right insert, asterisk indicates an unspecific cross-reacting band) and autophagy induction in non-infected and infected (48 hpi) DCs (silenced for all three proteins) was analysed by CytoID staining (CAT). Fluorescence profiles were overlaid to directly compare changes in autophagy. Data from three independent experiments (non-infected and 48 hpi) are summarised as bar graphs with arbitrary units **(B)**. The value obtained for non-infected cells was set to 1. **C)** Cells were fixed and costained for LC3 (red), chlamydial LPS (green) and DNA (blue) (top panel). Enlarged photographs in the second panel from top (I and II) show colocalisation between autophagy marker LC3 and chlamydial structures. The influence of siRNA silencing on the physical appearance of inclusions was visualised by DAPI (third panel from top). Enlarged photographs (lower panel, III and IV) display the size and structure of inclusions. **D)** The fluorescence intensity along a cellular cross section of interest (enlarged photographs I and II in **C**) was measured. Obtained profiles were overlaid and coloured. **E)** Triple siRNA-silenced infected and non-infected DCs (48 hpi) were analysed by flow cytometry using the IMAGEN kit. AllStars siRNA was used as control. Summarised data from three independent experiments **(F)** show the number of chlamydia-positive cells, as well as the bacterial load (MFI) of infected cells. The MFI values obtained for infected cell cultures (control siRNA) were set to 1 arbitrary unit. **G)** Triple-siRNA-silenced DCs were infected with chlamydia. DCs were cocultured with chlamydia-sensitised $CD8^+$ T cells for 48 h. AllStars siRNA, non-infected cells and CD3/CD28 beads were used as controls. IFN-γ secretion by $CD8^+$ T cells was assayed by ELISA. Relative values of IFN-γ secretion are expressed in arbitrary units (maximum value was set to 10) and are the means ± SD of three independent experiments. Statistical analysis in **B)**, **F)** and **G)** was performed as described in Methods (***$p < 0.001$; n=3).
